# Cardiac reverse remodeling in primary mitral regurgitation: mitral valve replacement vs. mitral valve repair

**DOI:** 10.1186/s12968-023-00946-9

**Published:** 2023-07-27

**Authors:** Thomas P. Craven, Pei G. Chew, Laura E. Dobson, Miroslawa Gorecka, Martine Parent, Louise A. E. Brown, Christopher E. D. Saunderson, Arka Das, Amrit Chowdhary, Nicholas Jex, David M. Higgins, Erica Dall’Armellina, Eylem Levelt, Dominik Schlosshan, Peter P. Swoboda, Sven Plein, John P. Greenwood

**Affiliations:** 1 Multidisciplinary Cardiovascular Research Centre & Department of Biomedical Imaging Science, Leeds Institute of Cardiovascular and Metabolic Medicine, University of Leeds, Leeds, LS2 9JT UK; 2 Department of Cardiology, Wythenshawe Hospital, Manchester University NHS Trust, Manchester, UK; 3 Philips, Guildford, England UK; 4 Leeds Teaching Hospitals NHS Trust, Leeds, UK

**Keywords:** Cardiovascular magnetic resonance, CMR, Mitral regurgitation, Mitral valve repair, Mitral valve replacement

## Abstract

**Background:**

When feasible, guidelines recommend mitral valve repair (MVr) over mitral valve replacement (MVR) to treat primary mitral regurgitation (MR), based upon historic outcome studies and transthoracic echocardiography (TTE) reverse remodeling studies. Cardiovascular magnetic resonance (CMR) offers reference standard biventricular assessment with superior MR quantification compared to TTE. Using serial CMR in primary MR patients, we aimed to investigate cardiac reverse remodeling and residual MR post-MVr vs MVR with chordal preservation.

**Methods:**

83 patients with ≥ moderate-severe MR on TTE were prospectively recruited. 6-min walk tests (6MWT) and CMR imaging including cine imaging, aortic/pulmonary through-plane phase contrast imaging, T1 maps and late-gadolinium-enhanced (LGE) imaging were performed at baseline and 6 months after mitral surgery or watchful waiting (control group).

**Results:**

72 patients completed follow-up (Controls = 20, MVr = 30 and MVR = 22). Surgical groups demonstrated comparable baseline cardiac indices and co-morbidities. At 6-months, MVr and MVR groups demonstrated comparable improvements in 6MWT distances (+ 57 ± 54 m vs + 64 ± 76 m respectively, p = 1), reduced indexed left ventricular end-diastolic volumes (LVEDVi; − 29 ± 21 ml/m^2^ vs − 37 ± 22 ml/m^2^ respectively, p = 0.584) and left atrial volumes (− 23 ± 30 ml/m^2^ and − 39 ± 26 ml/m^2^ respectively, p = 0.545). At 6-months, compared with controls, right ventricular ejection fraction was poorer post-MVr (47 ± 6.1% vs 53 ± 8.0% respectively, p = 0.01) compared to post-MVR (50 ± 5.7% vs 53 ± 8.0% respectively, p = 0.698). MVR resulted in lower residual MR-regurgitant fraction (RF) than MVr (12 ± 8.0% vs 21 ± 11% respectively, p = 0.022). Baseline and follow-up indices of diffuse and focal myocardial fibrosis (Native T1 relaxation times, extra-cellular volume and quantified LGE respectively) were comparable between groups. Stepwise multiple linear regression of indexed variables in the surgical groups demonstrated baseline indexed mitral regurgitant volume as the sole multivariate predictor of left ventricular (LV) end-diastolic reverse remodelling, baseline LVEDVi as the most significant independent multivariate predictor of follow-up LVEDVi, baseline indexed LV end-systolic volume as the sole multivariate predictor of follow-up LV ejection fraction and undergoing MVR (vs MVr) as the most significant (p < 0.001) baseline multivariate predictor of lower residual MR.

**Conclusion:**

In primary MR, MVR with chordal preservation may offer comparable cardiac reverse remodeling and functional benefits at 6-months when compared to MVr. Larger, multicenter CMR studies are required, which if the findings are confirmed could impact future surgical practice.

**Supplementary Information:**

The online version contains supplementary material available at 10.1186/s12968-023-00946-9.

## Introduction

Current guidelines on primary mitral regurgitation (MR) recommend mitral valve repair (MVr) over mitral valve replacement (MVR) whenever feasible [1, 2]. Supportive observational studies typically demonstrate worse early and long-term mortality post MVR [3, 4], however, numerous pre-date the routine use of chordal preservation techniques [3, 5, 6], which improves cardiac reverse remodeling post MVR [7–9]. Indeed, transthoracic echocardiographic (TTE) studies assessing left-ventricular (LV) reverse remodeling post MVr/MVR suggest it is comparable when chordal preservation is used [10, 11] and worse remodeling post-MVR when not [5, 6]. MVR is more frequently performed in older patients with more co-morbidities [12]. To date, no randomised trials have compared MVr and MVR in primary MR and studies using propensity matching in an attempt to overcome intrinsic bias present conflicting results [12, 13]. A randomised trial in ischaemic MR demonstrated comparable survival and LV reverse remodeling at 2 years, with greater recurrent MR and adverse events post MVr compared to post MVR with chordal preservation [14].

In primary MR, recurrent MR post-MVr is not uncommon, with moderate-severe MR reported in 13–17% in longitudinal studies [15, 16]. MVr typically results in equivalent [3, 12, 13] or more re-operations than MVR [17], however, the re-operation end-point may not account for all recurrent significant MR if patients are not amenable to, or deemed too high risk for, repeat surgery.

Accurate MR assessment is paramount to guide the need for surgical intervention and provide appropriate outcome comparisons between MVr/MVR. Cardiovascular magnetic resonance (CMR) is the reference standard for biventricular volume/function assessment [18] and compared to TTE, CMR MR quantification demonstrates superior reproducibility [19–21] and prognostication in primary MR [20, 22]. Additionally, CMR can accurately quantify the forward LV pump efficiency, despite the presence of severe MR, by assessing effective forward left ventricular ejection fraction (LVEF), calculated as aortic forward flow volume/left ventricular end diastolic volume (LVEDV) [23].

Ultimately, randomised trials comparing MVr vs MVR could guide future clinical decision making, especially in specific patient groups in whom MVR would not increase bleeding risk (patients in whom a tissue valve will last a lifetime or patients with a pre-existing indication for anticoagulation). Prior to this, rigorous hypothesis-generating observational data will be required; this study aimed to assess differences in cardiac reverse remodeling, residual MR (assessed by CMR) and functional capacity following surgical MVr and MVR with chordal preservation for primary MR, compared to a matched control group (moderate to/or severe MR patients on a watchful waiting pathway).

## Methods

### Study design

This single-center prospective observational cohort study recruited patients between February 2016 and February 2020 with primary MR at the Leeds Teaching Hospitals NHS Trust, Leeds, UK, during which time 313 patients had corrective surgery for MR, of which 168 patients without significant aortic valve disease had elective surgery for primary MR. All patients meeting inclusion/exclusion criteria were contacted, of which 83 were recruited. Inclusion criteria comprised moderate-severe or severe primary MR on echocardiography, aged > 18 years, suitable for elective surgical intervention, with capacity to provide written informed consent. Exclusion criteria comprised secondary (functional/ischaemic/atrial) MR, contraindications to CMR, significant (≥ moderate severity) aortic valve disease, uncontrolled atrial fibrillation (AF) > 120 bpm, New York Heart Association (NYHA) functional Class IV, terminal illness, haemodynamic instability, renal failure with an estimated glomerular filtration rate of < 30 ml/min/1.73m^2^, pregnancy or breast feeding, or inability to lie supine for 60 min.

At least moderate-severe MR was defined by an integrated echocardiographic assessment as per American Society of Echocardiography guidelines: vena contracta > 0.7cm^2^, proximal isovelocity surface area (PISA) radius > 0.8 cm, estimated regurgitant orifice area > 0.3cm^2^, MR regurgitant volume (Rvol) > 45 ml/beat, MR regurgitant fraction (RF) > 40% [24]. Surgical intervention (timing and technique) was decided by a multidisciplinary heart team, as per international guidance [1, 2], independent from the study. Pre-operative assessment commonly included transesophageal echocardiography and left ± right heart catheterisation where indicated. CMR imaging and 6-min walk tests (6MWT) were performed at baseline and 6-months post-surgery (MVR or MVr) or post-watchful waiting (control group). 6MWT followed American Thoracic Society guidelines [25]. Written informed consent was provided by all patients. The study was approved by local research ethics committee (Yorkshire & The Humber-South Yorkshire 15/YH/0503) and complied with the Declaration of Helsinki.

### CMR imaging

Baseline and 6-month follow-up CMR were performed (1.5T Philips Ingenia, Best, Netherlands). CMR protocol involved: (1) survey images, (2) LV short axis multi-slice, multi-phase cine imaging using a balanced steady-state free precession (bSSFP) sequence (TR 3 ms, TE 1.6 ms, flip angle 60°, SENSE factor 2, 10 mm thickness, 0 mm gap, in-plane spatial resolution 1.2 × 1.2 mm, 30 phases, matrix 192 × 131, typical FOV of 340 mm), (3) horizontal and vertical long axis cine imaging (4) transaxial right ventricular (RV) multi-slice, multi-phase bSSFP cine imaging (TR 2.8 ms, TE 1.41 ms, flip angle 60°, SENSE factor 1.8, 8 mm thickness, 0 mm gap, in-plane spatial resolution 1.88 × 1.88 mm, 20 phases, matrix 192 × 143, typical FOV 360 mm), (5) two orthogonal LV-outflow-tract and RV-outflow-tract views, (6) through-plane aortic and pulmonary phase contrast (PC)-CMR, planned at the aortic sinotubular junction, orthogonal to the aorta, to assess aortic flow and approximately 1 cm superior to the pulmonary valve, orthogonal to the main pulmonary artery to assess pulmonary flow. Velocity encoding was set to 150 cm/s and increased for repeat imaging if aliased. All PC sequences were planned with region of interest in the CMR scanner isocenter to reduce potential background phase-offset errors [26]. Other PC parameters: typical FOV 350 × 280 mm, TR 5.1 ms, TE 3.2 ms, flip angle 15°, temporal resolution 28 ms, number of signal averages 1, SENSE factor 2, turbo field echo factor 3, turbo field echo acquisition duration 30.8 ms, slice thickness 8 mm, 30 phases, phase percentage 100%, in-plane spatial resolution 2.5 × 2.5 mm, matrix 140 × 112, Cartesian sampling, and typical acquisition times 12–15 s for breath-held sequences. In patients with AF, two acquisitions of aortic/pulmonary PC-CMR imaging with the same parameters were obtained and the results averaged to account for heart rate variation, (7) Late-gadolinum enhancement (LGE) imaging, multi-slice LV-short axis covering base-apex (typical FOV 350 mm, 10 mm thickness, 0 mm gap) and (8) Modified Look-Locker inversion recovery sequence, single mid-ventricular short axis slice, pre and 15-min post gadolinium contrast (0.2 mmol/kg).

### CMR analysis

Images were analysed using commercially available software (cvi42, Circle Cardiovascular Imaging, Calgary, Alberta, Canada). CMR acquisition and analysis was overseen and performed respectively by a dedicated experienced member of the CMR research team. For continuity, volumetric and phase contrast flow analysis was performed by the same individual, strictly using the methodology described below. Biventricular endocardial contours were manually traced; the papillary muscles and trabeculations were considered part of the ventricular blood pool and volumes calculated by summation of disks [27]. For sequential CMR analysis, careful comparison with baseline images was ensured to optimise basal slice selection and contouring around any post-surgical artefact. Maximal left atrial (LA) volume was calculated using the bi-plane area-length method from horizontal and vertical long-axis cines during ventricular systole [28] and maximal right atrial (RA) area measured, inclusive RA appendage, from horizontal long-axis cines during ventricular systole [28, 29]. As per prior studies [20–22] and Society of Cardiovascular Magnetic Resonance recommendations [30], mitral and tricuspid regurgitation were quantified indirectly using the following formulas respectively: Mitral regurgitant volume (MR-Rvol) = LV stroke volume − aortic stroke volume and tricuspid regurgitant volume (TR-Rvol) = RV stroke volume − pulmonary stroke volume. Effective forward LVEF was calculated as: aortic forward flow volume/LVEDV [23]. LGE short-axis images were visually assessed for LGE, if present quantitative assessment was performed by semi-automated signal intensity analysis according to the full width at half maximum technique [31]. Myocardial T1 relaxation times (ms) were assessed via a region of interest at the mid ventricular septum, with extracellular volume fraction (ECV) calculated using the patient’s haematocrit via standard formula [32].

### Surgical technique

Surgical procedures were performed under general anaesthesia using a standard cardiopulmonary bypass technique via a 7–10 cm midline sternotomy incision and mild systemic hypothermia (30–34 °C). Intra-operative transesophageal echocardiography was utilised. Systemic heparinisation aorto-bicaval cannulation was performed. LA incision was made to expose and inspect the pathological mitral valve. Final decision to perform MVr or MVR was at discretion of the surgeon dependent upon feasibility of durable repair utilizing knowledge from prior imaging, heart team MDT discussions and intra-operative morphological assessment of the native valve/apparatus. All MVr were performed using Gore-Tex chordae sutures and supported by a Carpentier-Edwards Physio II annuloplasty ring (typical size 29–34 mm). MVR were performed using the St Jude mechanical valve, Edwards Perimount Magna bioprosthetic valve or St Jude Epic™ Mitral stented tissue valve (typical size 27–33 mm). At least partial chordal preservation was performed with MVR as routine practice. The type of prosthetic valve, preservation technique and suture placement technique were at the discretion of the surgeon. Mechanical MVR patients were treated with life-long anticoagulation (Vitamin K antagonist-warfarin) post-procedure. In selected cases AF was ablated with radiofrequency and coinciding LA appendage ligation performed.

### Statistical analysis

Data were analysed using SPSS version 26 (IBM Corp, Armonk, New York, USA). All continuous data were assessed for normality using Shapiro–Wilk test. Baseline, follow-up/residual and the changes from baseline to follow up variables were compared between the three groups (control/MVr/MVR). Continuous variables are expressed as mean ± standard deviation (SD) and categorical variables expressed as frequencies and percentages. Continuous data were assessed between all groups by one-way analysis of variance (normally distributed variables) or Kruskal–Wallis (non-normally distributed variables), both performed with Bonferroni post-hoc analysis on statistically significant variables for subgroup comparison. Categorical data were compared by Fisher’s exact test, if a significant difference was found between all groups, Fisher’s exact tests were performed between each group to assess inter-group differences. p < 0.05 was considered statistically significant. Additional subgroup analysis was performed using one-way analysis of variance or Kruskal–Wallis and Fisher’s exact test as above: (1) comparing follow-up variables between surgical groups after exclusion of patients with residual MR-RF ≥ 30%; (2) comparing all groups after excluding patients whom underwent concomitant coronary artery bypass grafting (CABG); (3) comparing baseline variables between surgical groups whom achieved an LVEF ≥ 50% vs LVEF < 50% at follow-up [27, 33] and (4) comparing baseline/change/follow-up variables between surgical groups with baseline LGE (LGE+) vs those without (LGE−). Indices of myocardial fibrosis (T1/ECV/LGE) compared between control/MVr/MVR at baseline and follow-up (including subgroup analysis excluding CABG cases) utilized cases with available paired baseline/follow-up data. Predictors of post-surgical remodeling and residual MR were calculated using a stepwise linear regression model with baseline variables entered as covariates. For regression analysis, all volumetric indices were indexed to body surface area including valve regurgitant volumes and missing T1/ECV/LGE data were dealt with by listwise deletion. Variables with a univariate p < 0.1 were included in multivariate analysis. Post-surgical LV-end-diastolic reverse remodeling is calculated as the percentage reduction in LVEDV from baseline to follow-up imaging [(Follow-up LVEDV − Baseline LVEDV)/Baseline LVEDV x − 1], with values inverted so positive values indicate increased/improved reverse remodeling.

## Results

**Fig. 1:**
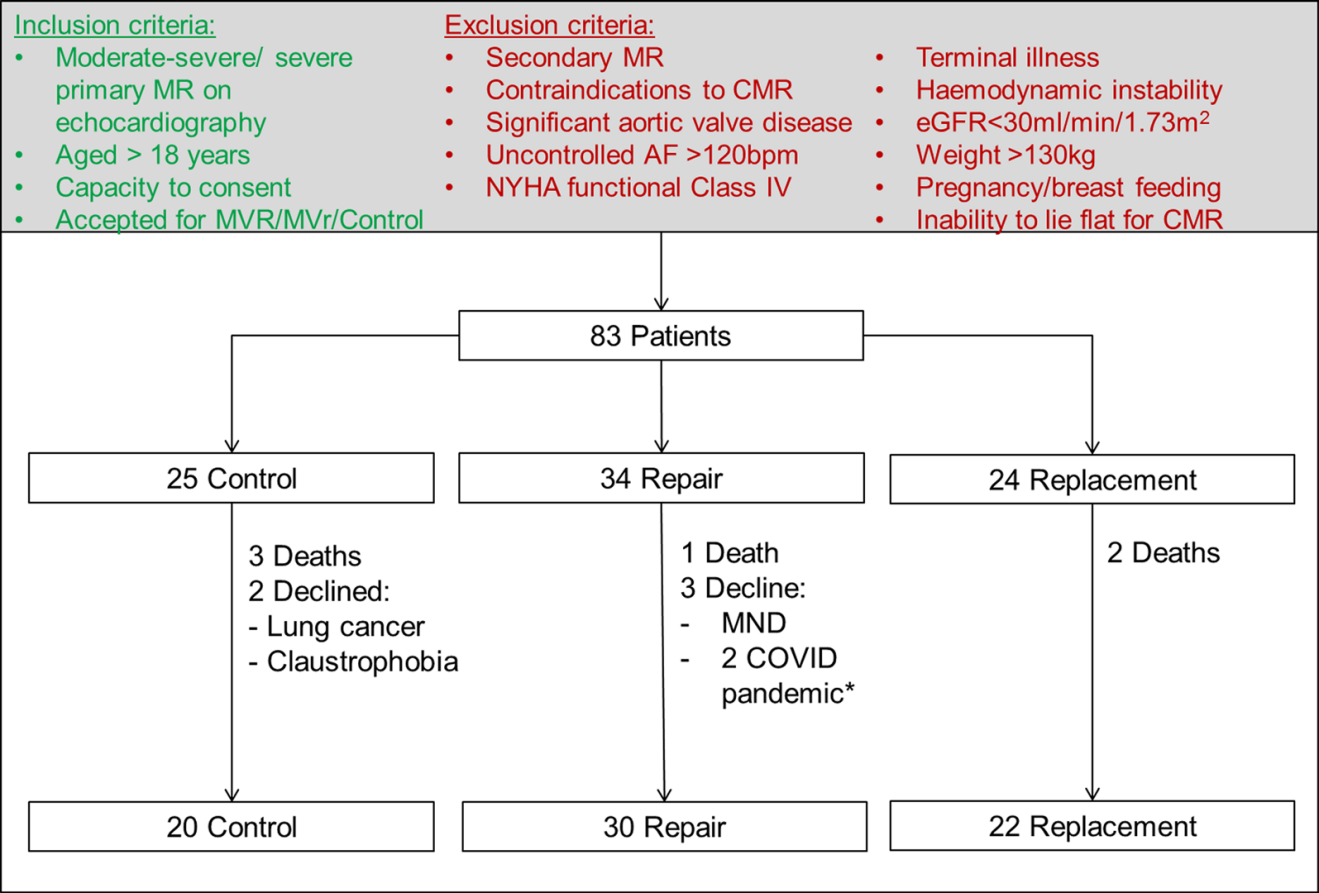
CONSORT patient flow diagram—a figure demonstrating number of patients recruited against the inclusion/exclusion criteria and numbers completing follow-up imaging by group. *2 patients declined follow up imaging due to the COVID-19 pandemic. AF: atrial fibrillation; CMR: cardiovascular magnetic resonance; eGFR: estimated glomerular filtration rate; MND: motor neurone disease; MR: mitral regurgitation; MVr: mitral valve repair; MVR: mitral valve replacement; NYHA: New York Heart Association

Eighty-three patients were recruited and scanned at baseline. By group, 34 patients underwent MVr (1 death and 3 patient dropouts: 1 developed motor neurone disease, 2 declined due to the COVID-19 pandemic); 24 underwent MVR (2 deaths); 25 controls were observed with watchful waiting (3 deaths and 2 patient drop-outs: 1 claustrophobic and 1 developed lung cancer). This resulted in 72 patients with paired CMR scans: 30 MVr, 22 MVR (14 mechanical, 8 bio-prosthetic valves) and 20 controls with follow-up CMR at 188 ± 27, 194 ± 25 and 233 ± 8 days respectively (Fig. 1, CONSORT flow diagram), with duration to follow-up CMR comparable between surgical groups (p = 1).

### Baseline patient characteristics

Baseline characteristics of the groups are presented in Table 1. There was no difference in age or sex between the groups. The underlying leaflet(s) affected differed between surgical groups (p = 0.014), with a greater proportion of posterior mitral valve leaflet (PMVL) disease in the MVr group and anterior mitral valve leaflet disease in the MVR group. Somewhat expectedly, the control group, compared with MVr and MVR groups, demonstrated a lower NYHA functional class (1.3 ± 0.6 vs 1.9 ± 0.7 and 2.2 ± 0.7 respectively, p = 0.001) and lower incidence of AF (20% vs, 53% and 59% respectively, p = 0.021). Otherwise, comorbidities and surgical risk scores (Log Euro/Log EuroII/ STS Mortality/morbidity) were comparable between groups.

**Table 1:** Baseline patient characteristics

		Control (n = 20)	Repair (n = 30)	Replace (n = 22)	P-values			
					All groups	Control vs Repair	Control vs Replace	Repair vs Replace
Male		11 (55%)	24 (80%)	16 (73%)	0.186			
Age (years)		64 ± 18	67 ± 11	66 ± 10	0.935			
Duration to follow-up (days)*		233 ± 8	188 ± 27	194 ± 25	0.001	0.001	0.008	1
BMI (kg/m^2^)		24.1 ± 3.3	26.2 ± 3.8	25.3 ± 5.0	0.275			
BSA (m^2^)		1.8 ± 0.2	1.9 ± 0.2	1.9 ± 0.2	0.414			
Systolic BP (mm/Hg)		125 ± 25	125 ± 15	125 ± 13	1			
Diastolic BP (mm/Hg)		73 ± 16	77 ± 13	77 ± 10	0.54			
Heart rate (bpm)		71 ± 10	72 ± 15	72 ± 13	0.885			
6MWT distance (m)		393 ± 118	365 ± 103	358 ± 79	0.485			
NYHA functional class								
I		15 (75%)	8 (27%)	4 (18%)	0.001	0.003	0.001	0.256
II		4 (20%)	16 (53%)	9 (41%)				
III		1 (5%)	6 (20%)	9 (41%)				
IV		0	0	0				
*Aetiology*								
Leaflet affected	PMVL	12 (60%)	26 (87%)	12 (54%)	0.027	0.149	0.332	0.014
	AMVL	1 (5%)	1 (3%)	5 (23%)				
	Bi-leaflet	7 (35%)	3 (10%)	5 (23%)				
Presence of flail leaflet		4 (20%)	8 (27%)	7 (32%)	0.703			
Surgical risk scores								
Log Euro		5.6 ± 4.7	4.7 ± 3.5	3.7 ± 2.4	0.736			
Log Euro II		1.5 ± 1.4	1.4 ± 1.0	1.6 ± 1.2	0.523			
STS mortality		1.5 ± 1.6	1.2 ± 1.2	1.9 ± 1.6	0.076			
STS mortality/morbidity		11.8 ± 7.1	9.5 ± 4.9	13.2 ± 5.9	0.053			
Comorbidities								
Smoking History		7 (35%)	14 (47%)	8 (36%)	0.713			
Diabetes mellitus		2 (10%)	1 (3%)	1 (5%)	0.679			
Hypertension		4 (20%)	11 (37%)	6 (27%)	0.486			
Atrial fibrillation		4 (20%)	16 (53%)	13 (59%)	0.021	0.022	0.014	0.781
Prior myocardial infarction		1 (5%)	0	1 (5%)	0.507			
Prior PCI		2 (10%)	0	1 (5%)	0.183			
Prior Stroke		1 (5%)	0	0	0.278			
Prior TIA		1 (5%)	1 (3%)	1 (5%)	1			
COPD		2 (10%)	1 (3%)	2 (9%)	0.599			
Chronic Kidney Disease		1 (5%)	0	1 (5%)	0.507			
Haemoglobin (g/L)		137 ± 11	143 ± 10	140 ± 14	0.15			
Creatinine (umol/L)		79 ± 14	81 ± 18	88 ± 20	0.244			

^*^Duration of time until repeat CMR imaging after either surgical intervention or baseline CMR in control group. 6MWT: 6-min walk test; AMVL: anterior mitral valve leaflet; BMI: body mass index; BP: blood pressure; BPM: beats per minute; BSA: body surface area; COPD: chronic obstructive pulmonary disease; NYHA: New York Heart Association; PCI: percutaneous coronary intervention; PMVL: posterior mitral valve leaflet; TIA: transient ischaemic attack; STS: society of thoracic surgeons

### Baseline CMR cardiac parameters

At baseline CMR assessment there were no significant differences in baseline biventricular volumes or quantified aortic, tricuspid, or pulmonary regurgitation between the groups (Table 2), except lower RVEF in the MVR and MVr groups when compared to controls at 46 ± 6.6% and 46 ± 9.4% vs 54 ± 8.0% respectively (p = 0.002). The control group had lower baseline quantitated MR volume compared to the MVr and MVR groups with an MR-Rvol of 49 ± 25 ml vs 66 ± 26 ml and 71 ± 29 ml (p = 0.002) and an MR-RF of 39 ± 13% vs 50 ± 10% and 52 ± 13% respectively (p = 0.001), resulting in a greater effective forward LVEF at 36 ± 8.3% vs 28 ± 7.6% and 27 ± 9.8 respectively (p = 0.002). There were no significant differences on baseline CMR between both surgical groups.

**Table 2:** Baseline CMR parameters

	Groups			P-values			
	Control (n = 20)	Repair (n = 30)	Replace (n = 22)	All groups	Control vs Repair	Control vs replace	Repair vs Replace
LVEDVi (ml/m^2^)	118 ± 25	124 ± 31	131 ± 27	0.332			
LVESVi (ml/m^2^)	50 ± 14	56 ± 20	61 ± 19	0.153			
LVSVi (ml/m^2^)	69 ± 14	68 ± 16	70 ± 13	0.85			
LVEF (%)	59 ± 5	55 ± 7.8	54 ± 8.1	0.173			
Effective forward-LVEF (%)	36 ± 8.3	28 ± 7.6	27 ± 9.8	0.002	0.008	0.003	1
LVMi (g/m^2^)	53 ± 13	62 ± 14	63 ± 18	0.063			
LA volume indexed (ml/m^2^)	85 ± 23	94 ± 31	107 ± 36	0.063			
MR Rvol (ml)	49 ± 25	66 ± 26	71 ± 29	0.002	0.012	0.004	1
MR RF (%)	39 ± 13	50 ± 9.7	52 ± 13	0.001	0.002	0.001	1
RVEDVi (ml/m^2^)	93 ± 24	94 ± 20	98 ± 17	0.429			
RVESVi (ml/m^2^)	43 ± 12	51 ± 14	54 ± 16	0.034	0.113	0.041	1
RVSVi (ml/m^2^)	52 ± 16	43 ± 10	44 ± 9.3	0.033	0.041	0.102	1
RVEF (%)	54 ± 8	46 ± 6.6	46 ± 9.4	0.002	0.004	0.005	1
AR Rvol (ml)	3.6 ± 3.8	4.2 ± 2.1	3.4 ± 2.4	0.111			
AR RF (%)	4.8 ± 3.9	6.8 ± 3.7	5.8 ± 4.1	0.106			
PR Rvol (ml)	2.3 ± 2.2	3.4 ± 3.3	2.5 ± 1.6	0.421			
PR RF (%)	2.9 ± 2.2	5.2 ± 5.5	3.9 ± 3.0	0.191			
TR Rvol (ml)	12 ± 16	16 ± 15	15 ± 13	0.385			
TR RF (%)	13 ± 14	19 ± 18	17 ± 15	0.353			
RAAi (cm^2^/m^2^)	14 ± 3	15 ± 3.8	15 ± 4.3	0.319			
Native T1 (ms)*	1017 ± 33	1029 ± 61	1040 ± 52	0.441			
ECV*	27.7 ± 3.4	27.1 ± 3.1	28.5 ± 2.8	0.351			
LGE presence**	5 (28%)	10 (37%)	10 (50%)	0.383			
Non-ischaemic	4 (22%)	7 (26%)	9 (45%)	0.546			
Ischaemic	1 (6%)	3 (11%)	1 (5%)				
LGE (%)**	1.0 ± 2.0	3.1 ± 4.4	3.5 ± 4.1	0.22			
LGE (g)**	1.1 ± 2.3	3.3 ± 4.9	3.9 ± 4.9	0.219			

^*^Analysis performed on patients with paired baseline/follow-up data (control, n = 16; repair, n = 23; replace, n = 20). **Analysis performed on patients with paired baseline/follow-up data (control, n = 18; repair, n = 27; replace, n = 20). AR: aortic regurgitation; ECV: extracellular volume fraction; EDV: end-diastolic volume; EF: ejection fraction; ESV: end-systolic volume; I: indexed to body surface area; LA: left atrial; LGE: late gadolinium enhanced myocardium; LV: left ventricular; LVM: left ventricular mass; MR: mitral regurgitation; PR: pulmonary regurgitation; RAA: right atrial area; RF: regurgitant fraction; Rvol: regurgitant volume; RV: right ventricular; SV: stroke volume; TR: tricuspid regurgitation

### Surgical variables

The operation variables are compared between surgical groups in Additional file 1. Thirty patients underwent MVr and twenty-two patients underwent MVR (Prosthesis: mechanical = 14, tissue = 8). MVr and MVR groups were comparable in terms of concomitant coronary artery bypass grafting (2 vs 2 respectively, p = 1.00), tricuspid valve repair (5 vs 2 respectively, p = 0.685) and AF ablations (1 vs 2 respectively, p = 0.567). Cardiopulmonary bypass time and cross-clamp times were comparable between the MVr and MVR groups at 124 ± 26 min vs 132 ± 47 min (p = 0.837) and 96 ± 28 min vs 94 ± 41 min (p = 0.333) respectively. After dividing the MVR group into those with direct MVR (n = 16) and those with MVR after an attempted repair (MVRar) (n = 6), the MVRar group had longer surgical procedure times compared to direct MVR and MVr groups, with a cardiopulmonary bypass time of 190 ± 32 min vs 111 ± 31 min and 124 ± 26 min (p = 0.001) and cross-clamp time of 146 ± 39 min vs 74 ± 19 min and 96 ± 28 min (p = 0.001) respectively. On sub-group analysis, direct MVR patients had comparable bypass but shorter cross clamp times compared to the MVr group at 111 ± 32 min vs 124 ± 26 min (p = 0.216) and 74 ± 19 min vs 96 ± 28 min (p = 0.046) respectively.

### Functional outcomes

Changes between the groups from baseline to follow up are presented in Table 3. At follow up, compared with controls, the MVr and MVR groups demonstrated improved 6MWT distances (+ 0.1 ± 55 m vs + 57 ± 54 m and + 64 ± 76 m respectively, p = 0.002) and significant improvement in NYHA functional class (p < 0.001), with no significant differences between both surgical groups in either outcome. At 6 months, there were no significant differences between all groups in 6MWT distances or NYHA functional class (Table 4).

**Table 3:** Change in functional, haemodynamic, and cardiac parameters from baseline to 6 month follow up assessment

	Groups			P-values			
	Control (n = 20)	Repair (n = 30)	Replace (n = 22)	All groups	Control vs repair	Control vs replace	Repair vs replace
Systolic BP (mmHg)	− 0.2 ± 21	+ 0.8 ± 11	+ 0.1 ± 12	0.952			
Diastolic BP (mmHg)	+ 0.5 ± 14	+ 2.8 ± 10	+ 0.1 ± 9.2	0.510			
Heart rate (bpm)	− 3.0 ± 10	+ 3.1 ± 21	− 1.8 ± 12	0.359			
6MWT distance (m)	+ 0.1 ± 55	+ 57 ± 54	+ 64 ± 76	0.002	0.007	0.005	1
NYHA functional class	0.15 ± 0.4	− 0.8 ± 0.7	− 1.1 ± 0.7	< 0.001	< 0.001	< 0.001	0.281
LVEDVi (ml/m^2^)	− 1.3 ± 12	− 29 ± 21	− 37 ± 22	< 0.001	< 0.001	< 0.001	0.584
LVESVi (ml/m^2^)	− 1.7 ± 7.4	− 4.0 ± 16	− 8.3 ± 18	0.360			
LVSVi (ml/m^2^)	− 0.1 ± 8.4	− 25 ± 15	− 28 ± 13	< 0.001	< 0.001	< 0.001	1
LVEF (%)	+ 0.4 ± 3.9	− 8.7 ± 8.9	− 8.8 ± 9.0	< 0.001	< 0.001	0.001	1
Effective forward-LVEF (%)	+ 0.2 ± 3.9	+ 8.8 ± 8.3	+ 14 ± 8.7	< 0.001	< 0.001	< 0.001	0.060
LVMi (g/m^2^)	+ 0.3 ± 4.3	− 3.8 ± 10	− 3.7 ± 11	0.256			
LA volume indexed (ml/m^2^)	+ 1.2 ± 19	− 27 ± 30	− 39 ± 26	< 0.001	0.002	< 0.001	0.545
MR Rvol (ml)	− 0.1 ± 12	− 47 ± 21	− 62 ± 27	< 0.001	< 0.001	< 0.001	0.064
MR RF (%)	+ 0.4 ± 7.0	− 29 ± 11	− 40 ± 14	< 0.001	< 0.001	< 0.001	0.002
RVEDVi (ml/m^2^)	− 0.9 ± 5.5	− 5.0 ± 16	− 7.1 ± 20	0.436			
RVESVi (ml/m^2^)	+ 0.6 ± 5.5	− 3.5 ± 14	− 9.1 ± 17	0.051			
RVSVi (ml/m^2^)	− 3.3 ± 9.0	− 1.5 ± 11	+ 1.9 ± 10	0.487			
RVEF (%)	− 0.8 ± 4.0	+ 1.0 ± 9.5	+ 4.9 ± 7.9	0.067			
TR Rvol (ml)	+ 0.5 ± 21	− 5.1 ± 17	− 2.9 ± 13	0.493			
TR RF (%)	+ 2.1 ± 21	− 6.6 ± 20	− 4.5 ± 14	0.614			
RAAi (cm^2^/m^2^)	0.0 ± 2.5	0.0 ± 2.9	− 1.1 ± 3.9	0.568			

Abbreviations as in Tables 1 & 2

**Table 4:** Residual functional, haemodynamic, and cardiac parameters at 6 month follow up assessment

	Groups			P-values			
	Control (n = 20)	Repair (n = 30)	Replace (n = 22)	All groups	Control vs repair	Control vs replace	Repair vs replace
Systolic BP (mmHg)	125 ± 14	126 ± 12	125 ± 15	0.975			
Diastolic BP (mmHg)	73 ± 10	80 ± 11	77 ± 11	0.134			
Heart rate (bpm)	68 ± 11	75 ± 15	71 ± 8.3	0.141			
6MWT distance (m)	393 ± 109	422 ± 82	422 ± 111	0.586			
NYHA (mean)	1.45 ± 0.7	1.1 ± 0.3	1.1 ± 0.3	0.087			
LVEDVi (ml/m^2^)	117 ± 28	94 ± 28	94 ± 25	0.005	0.011	0.016	1
LVESVi (ml/m^2^)	48 ± 15	52 ± 23	52 ± 20	0.863			
LVSVi (ml/m^2^)	69 ± 15	42 ± 9.3	42 ± 8.6	< 0.001	< 0.001	< 0.001	1
LVEF (%)	59 ± 5	47 ± 9.2	46 ± 8.1	< 0.001	< 0.001	< 0.001	1
Effective forward-LVEF(%)	36 ± 8.9	37 ± 8.7	41 ± 8.9	0.241			
LVMi (g/m^2^)	54 ± 11	59 ± 15	60 ± 17	0.307			
LA volume indexed (ml/m^2^)	86 ± 28	67 ± 37	69 ± 28	0.115			
MR Rvol (ml)	49 ± 23	19 ± 13	9.5 ± 7.0	< 0.001	< 0.001	< 0.001	0.088
MR RF (%)	39 ± 13	21 ± 11	12 ± 8.0	< 0.001	0.001	< 0.001	0.022
RVEDVi (ml/m^2^)	92 ± 24	89 ± 18	91 ± 20	0.875			
RVESVi (ml/m^2^)	44 ± 14	48 ± 13	45 ± 12	0.459			
RVSVi (ml/m^2^)	49 ± 15	42 ± 8.6	46 ± 11	0.093			
RVEF (%)	53 ± 8	47 ± 6.1	50 ± 5.7	0.011	0.01	0.698	0.224
TR Rvol (ml)	13 ± 17	11 ± 10	12 ± 9.0	0.628			
TR RF (%)	15 ± 20	13 ± 11	13 ± 8.8	0.809			
RAAi (cm^2^/m^2^)	14 ± 3	15 ± 3.6	14 ± 3.6	0.511			
Native T1 (ms)*	1012 ± 36	1044 ± 43	1042 ± 37	0.042	0.084	0.068	1
ECV (%)*	27.8 ± 2.2	27.4 ± 3.6	27.0 ± 2.9	0.888			
LGE presence**	7 (39%)	14(52%)	11 (55%)	0.634			
Non-ischaemic	5 (28%)	11(41%)	10 (50%)	0.698			
Ischaemic	2 (11%)	3 (11%)	1 (5%)				
LGE (%)**	1.7 ± 2.7	4.3 ± 4.9	3.2 ± 3.7	0.242			
LGE (g)**	1.9 ± 3.0	4.0 ± 4.3	3.0 ± 3.5	0.291			

^*^Analysis performed on patients with paired baseline/follow-up data (control, n = 16; repair, n = 23; replace, n = 20). **Analysis performed on patients with paired baseline/follow-up data (control, n = 18; repair, n = 27; replace, n = 20). Abbreviations as in Tables 1 and 2

### Cardiac reverse remodeling and quantitated valve regurgitation

**Fig. 2:**
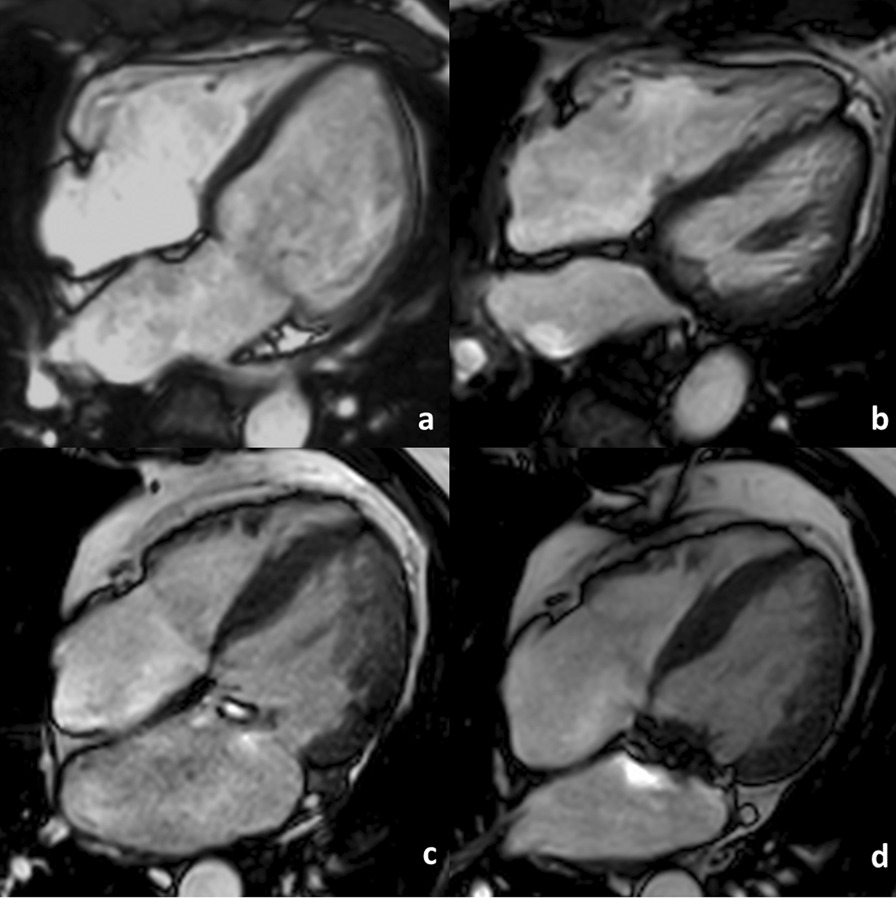
Comparison of cardiac reverse remodelling post mitral valve replacement vs repair: 4-chamber end-diastolic cine images acquired: **a** pre-mitral valve replacement, **b** post mitral valve replacement, **c** pre-mitral valve repair and **d** post-mitral valve repair

Changes to cardiac indices between baseline and follow up CMR are shown in Table 3 and the resultant residual cardiac indices are compared between groups in Table 4. Compared with controls, MVr and MVR resulted in comparable significant reductions in indexed LV end-diastolic volumes (− 1.3 ± 12 ml/m^2^ vs − 29 ± 21 ml/m^2^ and − 37 ± 22 ml/m^2^ respectively, p < 0.001), LVEF (+ 0.4 ± 3.9% vs − 8.7 ± 8.9% and − 8.8 ± 9.0% respectively, p < 0.001), indexed LA volumes (+ 1.2 ± 19 ml/m^2^ vs − 27 ± 30 ml/m^2^ and − 39 ± 26 ml/m^2^ respectively, p < 0.001) (Table 3) and improvements in effective forward LVEF (+ 0.2 ± 3.9% vs + 8.8 ± 8.3% and + 14 ± 8.7% respectively, p < 0.001). This resulted in lower LVEDVi (94 ± 28 ml/m^2^ and 94 ± 25 ml/m^2^ vs 117 ± 28 ml/m^2^ respectively, p = 0.005) and LVEF (47 ± 9.2% and 46 ± 8.1% vs 59 ± 5.0% respectively, p < 0.001) at 6-month follow-up in the MVr and MVR groups compared with controls and comparable effective forward LVEF between all groups (p < 0.241) (Table 4). There were no significant differences between surgical groups in terms of residual LV volumes/function or LA volume (Fig. 2). There were no significant differences between all groups in terms of change to RV volumes/function and RA areas, resulting in comparable residual right heart indices, except for lower residual RVEF in the MVr group compared with the controls (47 ± 6.1% vs 53 ± 8.0% respectively, p = 0.01). There was no significant difference in residual RVEF between MVr and MVR groups (47 ± 6.1% vs 50 ± 5.7% respectively p = 0.224). Both surgical groups demonstrated a significant reduction in and lower residual MR-Rvol and MR-RF compared with the control group (p < 0.001) (Tables 3 and 4). MVR resulted in a superior reduction in MR-RF (− 40 ± 14% vs − 29 ± 11%, p = 0.002), resulting in lower 6-month residual MR-RF compared with the MVr group (12 ± 8.0% vs 21 ± 11% respectively, p = 0.022). There were no significant differences between all three groups in changes to, or residual tricuspid regurgitation.

Importantly, the findings of comparable cardiac reverse remodeling between surgical groups but greater residual MR post-MVr remained present when patients with at least moderate [24] residual MR (MR-RF ≥ 30% on follow-up CMR) were excluded (Additional file 2). Subgroup analysis excluding patients with MR-RF ≥ 30% was performed comparing post-MVr (n = 24) vs post-MVR (n = 22), demonstrating comparable residual biventricular and bi-atrial volumes between both surgical groups with greater residual MR-RF post MVr than MVR (18 ± 8.2% vs 12 ± 8.0% respectively, p = 0.018). Separate sub-group analysis was performed with comparison of patients from all groups after patients who underwent concomitant CABGwere excluded (Additional file 3). Similarly, this resulted in no significant changes to the key findings.

Subgroup analysis comparing the baseline parameters of surgical patients who achieved follow-up LVEF ≥ 50% vs LVEF < 50% is presented in Additional file 4. There were no significant differences between co-morbidities, aetiology, functional or surgical parameters or tissues characteristics (Native T1/ECV/LGE) between the groups. Post-surgical patients with follow-up LVEF < 50% vs ≥ 50% had greater baseline LVEDVi (139 ± 29 ml/m^2^ vs 112 ± 23 ml/m^2^, p = 0.001), LVESVi (67 ± 21 ml/m^2^ vs 47 ± 12 ml/m^2^, p =  < 0.001), RVESVi (57 ± 13 ml/m^2^ vs 48 ± 15 ml/m^2^, p = 0.013) and MR severity (MR-RF: 54 ± 10% vs 47 ± 11%, p = 0.028) and lower baseline LVEF (52 ± 8.0% vs 59 ± 6.2%, p = 0.002).

### Predictors of cardiac reverse remodeling in surgical groups

**Fig. 3:**
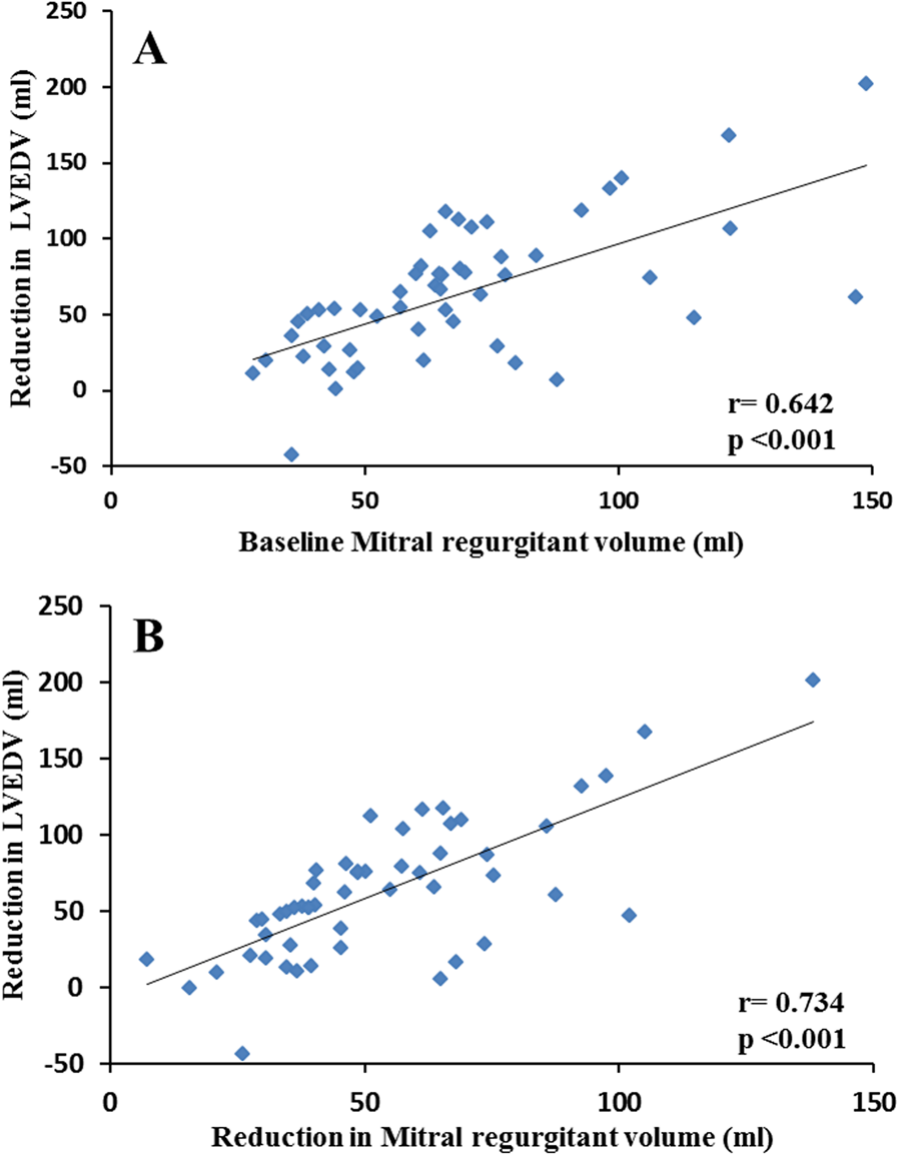
Pearson’s correlation of the reduction in left ventricular end-diastolic volume (LVEDV) post mitral valve correction with baseline mitral regurgitant volume (**A**) and the reduction in mitral regurgitant volume post mitral valve correction (**B**)

Baseline univariate and multivariate predictors of post-surgical LVEDV reverse remodeling are presented in Table 5 and predictors of follow-up indexed LVEDV, LVEF and MR-Rvol in Table 6. Baseline indexed MR-Rvol was the sole independent multivariate predictor of post-surgical LV-end-diastolic reverse remodeling. Baseline indexed LVEDV, MR-Rvol and NYHA were independent multivariate predictors of post-surgical indexed LVEDV. Baseline indexed LV end-systolic volume (LVESVi) was the sole independent multivariate predictor of post-surgical LVEF. Undergoing MVR (vs MVr) and baseline indexed left ventricular mass (LVMi) and LV stroke volume were independent multivariate predictors of indexed MR-Rvol at follow-up, with undergoing MVR (vs MVr) being the most significant (p < 0.001). Reductions in LVEDV post mitral valve correction demonstrate a moderate correlation with baseline MR-Rvol (r = 0.642, p < 0.001) and strong correlation with the reduction in MR-Rvol (r = 0.734, p < 0.001) (Fig. 3).

**Table 5:** Univariate and multivariate baseline predictors of post-surgical reverse remodeling of left ventricular end diastolic volume

Baseline parameters	Univariate				Multivariate			
	B ± Std Error	ß	95% CI	p	B ± Std Error	ß	95% CI	p
COPD	17.277 ± 9.091	0.260	− 0.983 to 35.538	0.063				NS
Haemoglobin (g/dL)	0.385 ± 0.177	0.294	0.030 to 0.741	0.034				NS
LVEDV (ml/m^2^)	0.059 ± 0.033	0.241	− 0.008 to 0.126	0.085				NS
LA-volume (ml/m^2^)	0.090 ± 0.032	0.368	0.025 to 0.155	0.007				NS
LVSV (ml/m^2^)	0.103 ± 0.061	0.232	− 0.019 to 0.225	0.097				NS
MR-volume (ml/m^2^)	0.520 ± 0.158	0.423	0.203 to 0.837	0.002	0.229 ± 0.073	0.405	0.082 to 0.375	0.003
Effective forward-LVEF (%)	− 0.567 ± 0.241	− 0.315	− 1.051 to -0.082	0.023				NS

Calculated as percentage change from baseline to follow-up from multiple linear regression analysis. All baseline variables with univariate p < 0.1 presented and utilized in multivariate analysis. All volumetric indices indexed to body surface area for accurate comparison. COPD: chronic obstructive pulmonary disease; EDV: end-diastolic volume; EF: ejection fraction; LA: left atrium; LV: left ventricle; MR: mitral regurgitation; SV: stroke volume

**Table 6:** Univariate and multivariate baseline predictors of post-surgical indexed LVEDV, LVEF and MR-volume at follow-up

Baseline parameters	Univariate				Multivariate			
	B ± Std Error	ß	95% CI	p	B ± Std Error	ß	95% CI	p
Left ventricular end-diastolic volume (indexed) at follow up								
CABG	− 23.503 ± 13.704	− 0.236	− 51.028 to 4.022	0.093				NS
AF	− 22.291 ± 6.877	− 0.417	− 36.105 to − 8.477	0.002				NS
NYHA class	− 12.776 ± 4.909	− 0.345	− 22.637 to − 2.915	0.012	− 8.167 ± 3.879	− 0.222	− 16.000 to − 0.333	0.041
Heart rate	− 0.687 ± 0.244	− 0.370	− 1.178 to − 0.197	0.007				NS
6MWT (m)	0.090 ± 0.040	0.300	0.009 ± 0.171	0.030				NS
LVEDV (ml/m^2^)	0.637 ± 0.091	0.703	0.454 ± 0.820	< 0.001	0.934 ± 0.162	1.011	0.605 to 1.262	< 0.001
LVESV ﻿(ml/m^2^)	0.844 ± 0.144	0.637	0.554 ± 1.134	< 0.001				NS
LVSV ﻿(ml/m^2^)	0.898 ± 0.211	0.515	0.474 ± 1.322	< 0.001				NS
LVEF (%)	− 0.984 ± 0.453	− 0.284	− 1.857 ± − 0.039	0.041				NS
LVM (g/m^2^)	0.909 ± 0.200	0.540	0.507 ± 1.312	< 0.001				NS
RVEDV ﻿(ml/m^2^)	0.705 ± 0.168	0.507	0.365 ± 1.046	< 0.001				NS
RVESV ﻿(ml/m^2^)	0.633 ± 0.234	0.357	0.163 ± 1.103	0.009				NS
RVSV ﻿(ml/m^2^)	1.134 ± 0.337	0.430	0.458 ± 1.810	0.001				NS
ECV(%)	2.830 ± 1.147	0.352	0.518 ± 5.142	0.018				NS
MR-Rvol ﻿(ml/m^2^)	0.798 ± 0.276	0.379	0.244 ± 1.352	0.006	− 0.995 ± 0.364	− 0.481	− 1.729 to − 0.260	0.009
AR-Rvol ﻿(ml/m^2^)	6.474 ± 3.588	0.247	− 0.731 ± 13.680	0.077				NS
Left ventricular ejection fraction at follow-up								
LVEDV ﻿(ml/m^2^)	− 0.153 ± 0.036	− 0.513	− 0.226 to − 0.080	< 0.001				NS
LVESV ﻿(ml/m^2^)	− 0.253 ± 0.050	− 0.579	− 0.354 to − 0.152	< 0.001	− 0.253 ± 0.050	− 0.579	− 0.354 to − 0.152	< 0.001
LVEF (%)	0.473 ± 0.141	0.430	0.191 to 0.755	0.001				NS
EF-LVEF (%)	0.421 ± 0.131	0.415	0.159 to 0.684	0.002				NS
LVM (g/m^2^)	− 0.231 ± 0.071	− 0.415	− 0.374 to − 0.087	0.002				NS
RVEDV ﻿(ml/m^2^)	− 0.125 ± 0.062	− 0.272	− 0.250 to 0.001	0.051				NS
RVESV ﻿(ml/m^2^)	− 0.139 ± 0.080	− 0.238	− 0.300 to 0.022	0.089				NS
MR-Rvol ﻿(ml/m^2^)	− 0.222 ± 0.093	− 0.320	− 0.409 to − 0.035	0.021				NS
AR-Rvol ﻿(ml/m^2^)	− 2.461 ± 1.171	− 0.285	− 4.813 to − 0.110	0.041				NS
TR-Rvol ﻿(ml/m^2^)	− 0.284 ± 0.156	− 0.250	− 0.597 to 0.029	0.074				NS
Mitral regurgitant volume (indexed) at follow-up								
MVR	− 4.511 ± 1.470	− 0.398	− 7.463 to − 1.559	0.003	− 4.861 ± 1.278	− 0.429	− 7.432 to − 2.291	< 0.001
Male	4.404 ± 1.773	0.332	0.844 to 7.965	0.016				NS
Heart rate	− 0.096 ± 0.054	− 0.246	− 0.204 to 0.011	0.079				NS
LVEDV ﻿(ml/m^2^)	0.075 ± 0.025	0.394	0.025 to 0.125	0.004				NS
LVESV ﻿(ml/m^2^)	0.079 ± 0.038	0.284	0.003 to 0.155	0.041				NS
LVSV ﻿(ml/m^2^)	0.142 ± 0.048	0.386	0.045 to 0.238	0.005	0.103 ± − 0.048	0.279	0.007 to 0.199	0.037
LVM (g/m^2^)	0.144 ± 0.046	0.406	0.052 to 0.236	0.003	0.100 ± 0.046	0.281	0.007 to 0.192	0.036
RVEDV ﻿(ml/m^2^)	0.078 ± 0.040	0.266	− 0.002 to 0.158	0.056				NS
RVESV ﻿(ml/m^2^)	0.095 ± 0.051	0.255	− 0.007 to 0.198	0.068				NS
MR-Rvol ﻿(ml/m^2^)	0.170 ± 0.058	0.383	0.053 to 0.286	0.005				NS

All baseline variables with univariate p < 0.1 presented and utilized in multivariate analysis. All volumetric indices indexed to body surface area for accurate comparison. 6MWT: 6-min walk test distance; AR: aortic regurgitation; CABG: coronary artery bypass grafting; ECV: extracellular volume fraction; EDV: end-diastolic volume; EF-LVEF: effective forward left ventricular ejection fraction; ESV: end-systolic volume; i: indexed to body surface area; LA: left atrial; LV: left ventricular; LVEF: left ventricular ejection fraction; LVM: left ventricular mass; MR: mitral regurgitation; MVR: mitral valve replacement; Rvol: regurgitant volume; NYHA: New York Heart Association; RV: right ventricular; SV: stroke volume; TR: tricuspid regurgitation

### Myocardial fibrosis

The CMR protocol was generally well tolerated and of good quality however T1/LGE sequences were incomplete or insufficient quality in some cases. Sufficient quality baseline T1 & LGE images were acquired in all controls, 25 and 29 MVr respectively and 20 MVR. After follow up, available paired baseline/follow-up indices of LGE (control, n = 18; MVr, n = 27; MVR, n = 20) and T1/ECV (control, n = 16; MVr, n = 23; MVR, n = 20) demonstrated no significant difference between groups at baseline or follow-up, Tables 2 and 4 respectively. The single MVr with insufficient baseline image quality for LGE quantification, detailed above, was sufficient to determine presence/type of LGE. Therefore, 50 surgical patients (30 MVr, 20 MVR) were available for subgroup analysis comparing baseline LGE+ (n = 21) vs LGE− (n = 29) demonstrating comparable baseline, change and follow-up volumetric and functional parameters (Additional file 5), except for a lower RVEF in LGE+ vs LGE− (42 ± 6.0% vs 47 ± 8.9% respectively, p = 0.024). Finally, as demonstrated above (Tables 5 and 6), indices of myocardial fibrosis at baseline were not independently predictive of post-surgical change in LVEDV or follow-up indexed LVEDV, MR-RVol or LVEF after multiple linear regression.

## Discussion

To our knowledge, this is the first CMR study comparing cardiac reverse remodeling and quantified residual MR between MVr and MVR, using a longitudinal control group for comparison. Importantly, at baseline the study had naturally well-matched surgical groups, with no significant differences in cardiac parameters and co-morbidities. The study has five important findings: 1, both MVr and MVR resulted in comparable LV reverse remodeling and functional improvement; 2, RVEF was worse post-MVr vs controls compared to post-MVR; 3, MVR was the most significant predictor of lower residual MR, resulting in a greater reduction in and lower residual MR than MVr; 4, the cardiac reverse remodeling and residual MR findings post-surgery remained unchanged on subgroup analysis when those with at least moderate residual MR were excluded and separate subgroup analysis with patients undergoing concomitant CABG excluded and 5, baseline LVESVi was the sole multivariate predictor of LVEF post mitral surgery.

### LV reverse remodeling

Our LV reverse remodeling findings demonstrate equivalency between MVr and MVR. These findings are in keeping with prior echocardiographic studies [10, 11, 34] and the only prior CMR study [23] to directly compare remodeling between MVr and MVR with chordal preservation at 3-months (n = 20) and 27-months (n = 14). Like previous studies [10, 11, 35], we demonstrated a significant decrease in LVEF post-operatively, finding no significant difference between surgical groups. Compared with baseline, an initial reduction in LVEF is common post mitral valve correction [5, 10, 11, 23, 36–38] and then can improve for up to 3–4 years post-mitral valve correction before plateauing [38]. In significant mitral regurgitation, the LV offloads into the lower-pressure/more-compliant left atrium resulting in an augmented LVEF. However, the LV is less efficient, with less blood appropriately leaving via the aortic valve, as evidenced by reduced effective-forward-LVEF. Therefore, although LVEF reduces initially post-operatively, the LV becomes more efficient as evidenced by improved post-surgical effective-forward-LVEF in this and previous studies [23]. However, we demonstrate, somewhat expectedly, patients with greater MR severity, pre-surgical adverse remodeling and lower LVEF at baseline demonstrate poorer LVEF at 6-months post mitral valve correction. In comparison with prior CMR studies [23, 37, 39], our surgical cohorts demonstrate greater baseline MR severity (with definitively severe mean CMR quantified MR) and/or LV dilatation. Prior observations potentially explain the variability between cohorts. Uretsky et al. [37] discussed that several observational studies noted patients who underwent mitral correction for echocardiography derived significant MR did not have severe MR on further assessment (with a significant percentage having mild MR) [19, 37, 40], with echocardiography’s comparative suboptimal inter/intra-observer variability and potential to overestimate MR severity deemed potentially contributory [19, 20, 22, 37, 41–43].

In keeping with prior research, we demonstrate baseline MR-Rvol as the most significant independent predictor of LV-end-diastolic reverse remodeling, showing a good correlation with reduction in LVEDV post mitral valve correction [37] and is also an independent predictor of post-surgical LVEDVi. We also found that patients with greater baseline MR-Rvol understandably have a greater reduction in MR-Rvol post-correction, resulting in greater LV reverse remodeling and lower residual LVEDVi. Additionally, we found patients with greater baseline LVMi prone to more residual MR. Increases in LVM occur as an adverse remodeling response to chronic LV volume overload in primary MR [36, 44]. Prior transthoracic echocardiography (TTE) studies assessing recurrent MR demonstrate numerically (but non-statistically significant) greater baseline LVMi in patients that develop recurrent primary MR post MVr [15, 45]. As CMR provides reference standard LVM and MR quantification, the superior accuracy afforded may demonstrate significant results that prior TTE studies could not. Larger CMR studies are now required to assess the reproducibility and significance of this finding. Reinforcing prior research [46], our findings demonstrate baseline LVESVi as the sole multivariate predictor of post-surgical LVEF at follow-up. This finding supports current international guidelines recommending surveillance of LV-end systolic dimensions/volumes in asymptomatic primary MR patients and its use to guide patient selection for early intervention. Further studies to clarify CMR thresholds for early intervention are now required.

### RV reverse remodeling

In keeping with Uretksy et al*.* we found no significant change in indexed RVEDV/RVESV post-MVr/MVR [19, 37]. Two CMR studies by Uretsky et al., the majority of which had MVr, demonstrated no statistically significant change between pre- and post-operative RVEF. Unfortunately, small numbers of MVR operations resulted in no direct comparison between surgical techniques. Our study demonstrated lower RVEF post-MVr vs controls (p = 0.01), but no significant difference between the two surgical groups on direct comparison (p = 0.224). However, our MVr group underwent a proportionally greater number of tricuspid valve repairs compared to the MVR group (5 vs 2 respectively), which may have blunted the RVEF augmentation in the MVr group. There were, however, no significant differences in the quantified tricuspid regurgitant fraction between the groups pre-operatively or at follow-up to support this. Therefore, the lower RVEF in the MVr group vs controls may be because of a lower reduction in and greater residual MR-RF compared with the MVR group.

### Differences in residual MR between surgical groups

MVR compared to MVr resulted in a greater reduction in MR-RF post-operatively and hence lower residual MR-RF. This finding is demonstrated on inter-group comparison and reinforced with regression analysis whereby undergoing MVR (vs MVr) was the most significant predictor of having less residual MR. Our findings of greater residual MR post-MVr are in keeping with prior echocardiographic studies [11, 47]. Importantly our central findings remain unchanged on subgroup analysis with direct comparison between surgical groups (Additional file 2) even after exclusion of patients with ≥ moderate residual MR.

### Myocardial fibrosis

Our study demonstrates LGE prevalence comparable between groups and with cohorts of severe primary MR patients in prior studies, similarly demonstrating reduced baseline RVEF in patients with LGE + vs LGE− [48, 49]. Otherwise, we found no significant differences in volumetric/functional indices at rest/change/follow-up between LGE+ vs LGE−. Although mean baseline/follow-up LVEF was 5% lower in LGE+ vs LGE−, this was not statistically significant. Additionally on multiple linear regression, LGE presence was not shown to be an independent predictor of the change in LVEDVi or follow-up LVEDVi or LVEF. Prior studies vary, demonstrating reduced [49] and comparable LVEF between LGE+ vs LGE− MR patients [48]. Given LGE presence in MR patients correlates with MR severity and LV dilatation [48] and LVEF artificially increases with increasing MR severity [50, 51], this may impact results when comparing LVEF between LGE+ vs LGE− MR patients. However, as RVEF commonly decreases with increasing MR severity [50, 52] and CMR provides reference standard assessment [18], it is not unexpected that LGE+ MR patients demonstrate lower RVEF vs LGE− reproducibly across studies, whilst LVEF varies [48, 49], whereas mitral valve prolapse patients with ≤ mild MR demonstrate comparable RVEF and impaired LVEF in LGE+ vs LGE− patients [53].

## Study strengths

To date, our study may provide the most accurate comparison of cardiac reverse remodeling and quantification of residual MR between MVr/MVR in primary MR by the use of CMR and naturally well-matched surgical groups at baseline.

CMR is the reference standard for biventricular assessment [18] and arguably more accurate at assessing MR severity than TTE [19, 20, 22, 37]. This disparity may increase post-operatively, as acoustic shadow artefacts from mitral prostheses can restrict MR assessment by TTE [54]. Guidelines therefore recommend combined transthoracic/transesophageal echocardiography for accurate prosthetic MR assessment [54], potentially reducing the accuracy of studies solely utilising TTE to compare residual MR between surgical groups. Using CMR, prosthesis-related distortions of the magnetic field can create the potential for volume and flow miscalculation. However, this was mitigated with consistent LV basal slice analysis and indirect MR quantification (LV-Aortic stroke volume method), as aortic PC-CMR, planned carefully to avoid artefact, increases the distance from the prosthesis and accuracy of volume/flow assessment [55].

Baseline cardiac indices, surgical risk scores and co-morbidities were similar between surgical groups, potentially minimising bias. To expand upon prior studies comparing MVr vs MVR [12, 13, 23], we examined indices of myocardial fibrosis, demonstrating parity between the groups, thereby providing additional evidence of minimal baseline bias between surgical groups. Patients undergoing MVR are typically older with more comorbidities than those referred for MVr. In primary MR, propensity matched studies performed to overcome this bias present conflicting results, with Gilinov et al.demonstrating no significant difference in long term survival and freedom from re-operation between MVr and MVR with chordal preservation [12], whilst Lazam et al. found lower operative mortality, better long term survival and fewer valve related complications post MVr, specifically in patients with flail leaflets [13].

### Clinical implications

Perhaps controversially, our findings of comparable cardiac reverse remodeling following MVr and MVR and lower residual MR-RF post-MVR, could pose a potential challenge to the current recommendation of ‘repair whenever feasible’. However, this would need to be confirmed in much larger multi-center series. Given cardiopulmonary bypass time and cross-clamp time both correlate with post-operative mortality and morbidity [56, 57], relaxing the recommendations in selected cases (e.g. in patients with another indication for lifelong anticoagulation or in older patients where a tissue valve may last a lifetime) may not adversely affect cardiac reverse remodeling and might positively impact surgical outcomes. As MVR is arguably more durable, with less recurrent MR [11, 47] then our results, if replicated in larger studies or randomised trials could impact clinical practice.

Optimising the timing to intervene for asymptomatic primary MR is challenging. Our findings, that baseline LVESVi was the sole multivariate predictor of post-surgical LVEF, reinforces current international guidance advising monitoring cardiac remodeling by assessing the LV at end-systole [1, 2]. Further CMR studies are required to derive prognostic CMR thresholds to supplement decisions on early intervention in primary MR.

## Study limitations

This was a single center, non-randomised, prospective observational study with the potential for bias as with all studies of this design, therefore larger multicenter studies are required to validate the findings. We specifically recruited patients with primary MR and those undergoing elective surgery, therefore our results may not be generalizable to those with secondary MR or undergoing emergency surgery. As a non-randomised study intrinsic baseline differences between the groups could not be controlled. However, as demonstrated in Table 1, there were no significant differences between the surgical groups in terms of age, sex, or comorbidities. Despite differences in the underlying leaflet pathology between surgical groups there was no significant difference in cardiac reverse remodeling. The group sizes are modest by comparison with prior longitudinal MVR and MVr outcome studies, however the use of CMR and its high reproducibility for volumes [18] and flow quantification [19–21] means that much smaller sample sizes are required to detect a change compared to standard TTE. Except for 2 patients who had complete chordal preservation with MVR, partial chordal preservation was performed as routine practice in our study; however, complete chordal preservation is the optimal technique [8], which if performed may have led to greater remodeling differences between the groups, potentially in favour of MVR. Six patients in the MVr group had residual MR-RF ≥ 30%, potentially biasing results against the MVr group, however our central findings comparing MVr and MVR remained unchanged following subgroup analysis after exclusion of these patients. T1/LGE sequences were incomplete/insufficient image quality in some cases, resulting in analysis on a reduced cohort, potentially introducing bias/reducing accuracy. Finally, reverse remodeling can potentially continue until 3–4 years post mitral valve correction [38] and our study specifically assessed cardiac reverse remodeling and functional changes after 6-months, so the study is unable to confirm that residual differences between surgical groups would result in different long term clinical outcomes.

There were differences in leaflets affected between groups, with the MVr group more typically having PMVL disease than the other groups. This is unsurprising given PMVL prolapse is more amenable to successful surgical repair [58] and international guidelines advise repair whenever feasible [1, 2], making this difficult to control for in an observational study. At baseline, compared to the watchful waiting control group, both surgical groups demonstrated worse NYHA functional class, quantitated MR, RVEF and had a greater proportion of patients in AF, demonstrating as expected that the surgical groups were at a more advanced stage on the MR severity spectrum.

## Conclusion

In primary MR, MVR with chordal preservation may offer comparable cardiac reverse remodeling benefits at 6-months compared to MVr. Larger, multicenter CMR studies are required, which if the findings are confirmed, may have implications for future surgical practice.

## Supplementary Information

**Additional file 1. **Operation variable comparisons between surgical groups. Surgical variables between groups and differences between groups when mitral valve replacement group was divided into those that received direct replacement and had mitral valve replacement after attempted repair.

**Additional file 2. **Subgroup analysis of surgical groups after exclusion of patients with moderate residual MR. Subgroup analysis of follow up cardiac, haemodynamic and functional indices between mitral valve repairand mitral valve replacement after excluding cases with at least moderate residual mitral regurgitation.

**Additional file 3. **Subgroup analysis with CABG patients excluded. Subgroup analysis comparing groups after exclusion of patients who underwent CABG: demonstrating the baseline patient characteristics (Table S3), baseline CMR parameters (Table S4), change in functional, haemodynamic and cardiac parameters from baseline to 6 month follow up assessment (Table S5) and residual functional, haemodynamic and cardiac parameters at 6 month follow up assessment (Table S6).

**Additional file 4. **Subgroup analysis of surgical groups—Comparison of baseline parameters of surgical patients when divided into groups by follow-up left ventricular ejection fraction. Subgroup analysis of surgical groups demonstrating baseline parameters between those achieving LVEF ≥ 50% vs LVEF < 50% at follow-up (Table S7).

**Additional file 5. **Subgroup analysis of surgical groups by baseline presence or absence of late gadolinium enhanced myocardium. Subgroup analysis of surgical groups comparing those with and without late gadolinium enhancement at baseline—baseline patient characteristics and surgical parameters (Table S8) and baseline, change and follow-up CMR and functional parameters (Table S9).

## Data Availability

The datasets used and/or analysed during the current study are available from the corresponding author on reasonable request. Dr. Seth Uretsky served as the *JCMR* Guest Editor for this manuscript.

## Abbreviations

6MWT: 6-Minute walk test
AF: Atrial fibrillation
AMVL: Anterior mitral valve leaflet
CMR: Cardiovascular magnetic resonance
ECV: Extracellular volume
EDV: End-diastolic volume
EF: Ejection fraction
ESV: End-systolic volume
I: Indexed to body surface area
LA: Left atrium
LGE: Late gadolinium enhancement
LV: Left ventricle
MR: Mitral regurgitation
MVr: Mitral valve repair
MVR: Mitral valve replacement
MVRar: Mitral valve replacement after attempted repair
NYHA: New York Heart Association
PC: Phase contrast
PMVL: Posterior mitral valve leaflet
RA: Right atrium
RF: Regurgitant fraction
RV: Right ventricle
R-vol: Regurgitant volume
TR: Repetition time
TTE: Transthoracic echocardiography
