# Supplementary material for: Cardiac reverse remodeling in primary mitral regurgitation: mitral valve replacement vs. mitral valve repair
Source: J Cardiovasc Magn Reson. 2023 Jul 27;25:43. doi: 10.1186/s12968-023-00946-9 (PMC10373289; doi:10.1186/s12968-023-00946-9)
Supplement: Supplementary file 1 — Additional file 1. Operation variable comparisons between surgical groups. Surgical variables between groups and differences between groups when mitral valve replacement group was divided into those that received direct replacement and had mitral valve replacement after attempted repair. [file 12968_2023_946_MOESM1_ESM.docx]

Additional File 1

Table S1. Operation variable comparisons between surgical groups

| Surgical variable | Repair (n=30) | Replace (Tissue = 8,  Mechanical = 14) | | P-Value(s) | | | |
| --- | --- | --- | --- | --- | --- | --- | --- |
| CABG | 2 (7%) | 2 (9%) | | 1 |  | | |
| AF ablation | 1 (3%) | 2 (9%) | | 0.567 |  |  |  |
| Tricuspid valve repair | 5 (17%) | 2 (9%) | | 0.685 |  |  |  |
| Bypass duration (min ± SD) | 124±26 | 132±47 | | 0.837 |  |  |  |
| Cross clamp time (min. ± SD) | 96±28 | 94±41 | | 0.333 |  |  |  |
|  | Repair (n=30) | Attempted repair? | | All groups | MVr vs MVR | MVr vs MVRar | MVR vs MVRar |
|  |  | No (n=16) | Yes (n=6) |  |  |  |  |
| Bypass duration (min ± SD) | 124±26 | 111±31 | 190±32 | 0.001 | 0.216 | <0.001 | 0.012 |
| Cross clamp time (min ±SD) | 96±28 | 74±19 | 146±39 | 0.001 | 0.046 | 0.071 | 0.001 |

**Table S1 legend:** Surgical variables between groups and differences between groups when mitral valve replacement group divided into those that received direct replacement and had mitral valve replacement after attempted repair (MVRar).

Abbreviations: AF, atrial fibrillation; CABG, coronary artery bypass grafting; MVr, mitral valve repair; MVR, mitral valve replacement; MVRar, mitral valve replacement after attempted repair, SD standard deviation.
