# Supplementary material for: Cardiac reverse remodeling in primary mitral regurgitation: mitral valve replacement vs. mitral valve repair
Source: J Cardiovasc Magn Reson. 2023 Jul 27;25:43. doi: 10.1186/s12968-023-00946-9 (PMC10373289; doi:10.1186/s12968-023-00946-9)
Supplement: Supplementary file 2 — Additional file 2. Subgroup analysis of surgical groups after exclusion of patients with moderate residual MR. Subgroup analysis of follow up cardiac, haemodynamic and functional indices between mitral valve repairand mitral valve replacement after excluding cases with at least moderate residual mitral regurgitation. [file 12968_2023_946_MOESM2_ESM.docx]

**Additional File 2 -**

# Table S2 – Subgroup analysis of follow up cardiac, haemodynamic, and functional indices between surgical groups after excluding cases with at least moderate residual mitral regurgitation

|  | Repair | Replace | P-value |
| --- | --- | --- | --- |
|  | (n=24) | (n=22) |  |
| Systolic BP (mmHg) | 125±13 | 125±15 | 0.851 |
| Diastolic BP (mmHg) | 79±10 | 77±11 | 0.584 |
| Heart rate (bpm) | 77±16 | 71±8.3 | 0.131 |
| 6MWT distance (m) | 412±77 | 422±111 | 0.261 |
| NYHA (mean) | 1.0±0.2 | 1.1±0.3 | 0.467 |
| LVEDVi (ml/m^2^) | 89±26 | 94±25 | 0.442 |
| LVESVi (ml/m^2^) | 49±22 | 52±20 | 0.598 |
| LVSVi (ml/m^2^) | 40±8.6 | 42±8.6 | 0.505 |
| LVEF (%) | 47±9.2 | 46±8.1 | 0.641 |
| Effective forward LVEF (%) | 38±8.3 | 41±8.9 | 0.384 |
| LVMi (g/m^2^) | 57±14 | 60±17 | 0.527 |
| LA volume indexed (ml/m^2^) | 60±30 | 69±28 | 0.194 |
| MR Rvol (ml) | 14±8.4 | 9.5±7.0 | 0.068 |
| MR RF (%) | 18±8.2 | 12±8.0 | 0.018 |
| RVEDVi (ml/m^2^) | 86±17 | 91±20 | 0.356 |
| RVESVi (ml/m^2^) | 45±10 | 45±12 | 0.965 |
| RVSVi (ml/m^2^) | 41±8.9 | 46±11 | 0.102 |
| RVEF (%) | 48±5.4 | 50±5.7 | 0.103 |
| TR Rvol (ml) | 10±11 | 12±9.0 | 0.217 |
| TR RF (%) | 12±11 | 13±8.8 | 0.454 |
| RAAi (cm^2^/m^2^) | 14±3.6 | 14±3.6 | 0.675 |
| Native T1 (ms)* | 1046±46 | 1042±37 | 0.641 |
| ECV (%)* | 27.4±3.1 | 27.0±2.9 | 0.566 |
| LGE presence:** | 13 (59%) | 11 (55%) | 1 |
| Non-ischaemic | 10 (45%) | 10 (50%) | 0.810 |
| Ischaemic | 3 (14%) | 1 (5%) |  |
| LGE (%)** | 4.9±5.0 | 3.2±3.7 | 0.746 |
| LGE (g)** | 4.5±4.7 | 3.0±3.5 | 0.780 |

*analysis performed on patients with paired baseline/follow-up data (repair, n=19; replace, n=20). ** analysis performed on patients with paired baseline/follow-up data (repair, n=22; replace, n=20). Data are mean ± standard deviation. Abbreviations: AR, aortic regurgitation; ECV, extracellular volume; EDV, end-diastolic volume; EF, ejection fraction; ESV, end- systolic volume; i, indexed to body surface area; LA, left atrial; LGE, late gadolinium enhanced myocardium; LV, left ventricular; LVM, left ventricular mass; MR, mitral regurgitation; PR, pulmonary regurgitation; RAA, right atrial area; RF, regurgitant fraction; Rvol, regurgitant volume; RV, right ventricular; SV, stroke volume; TR, tricuspid regurgitation.
