# Supplementary material for: Cardiac reverse remodeling in primary mitral regurgitation: mitral valve replacement vs. mitral valve repair
Source: J Cardiovasc Magn Reson. 2023 Jul 27;25:43. doi: 10.1186/s12968-023-00946-9 (PMC10373289; doi:10.1186/s12968-023-00946-9)
Supplement: Supplementary file 3 — Additional file 3. Subgroup analysis with CABG patients excluded. Subgroup analysis comparing groups after exclusion of patients who underwent CABG: demonstrating the baseline patient characteristics (Table S3), baseline CMR parameters (Table S4), change in functional, haemodynamic and cardiac parameters from baseline to 6 month follow up assessment (Table S5) and residual functional, haemodynamic and cardiac parameters at 6 month follow up assessment (Table S6). [file 12968_2023_946_MOESM3_ESM.docx]

**Additional file 3. Table S3. Subgroup analysis with CABG patients excluded: Baseline**

# patient characteristics

|  | |  |  |  | P-values | | | |
| --- | --- | --- | --- | --- | --- | --- | --- | --- |
|  |  | Control  (n=20) | Repair  (n=28) | Replace  (n=20) | All groups | Control vs  Repair | Control vs  Replace | Repair vs  Replace |
| Male (%) |  | 11 (55) | 23 (82) | 14 (70) | 0.134 |  | | |
| Age (years) |  | 64±18 | 66±10 | 66±10 | 0.996 |  |  |  |
| Duration to follow-up (days)* | | 233±8 | 188±27 | 194±26 | 0.001 | 0.002 | 0.002 | 1 |
| BMI (kg/m^2^) |  | 24.1±3.3 | 25.9±4.1 | 25.6±5.0 | 0.377 |  | | |
| BSA (m^2^) |  | 1.8±0.2 | 1.9±0.2 | 1.9±0.2 | 0.397 |  |  |  |
| Systolic BP (mm/Hg) | | 125±25 | 125±14 | 124±14 | 1 |  |  |  |
| Diastolic BP (mm/Hg) | | 73±16 | 77±13 | 76±10 | 0.512 |  |  |  |
| Heart rate (bpm) | | 71±10 | 72±15 | 72±13 | 0.904 |  |  |  |
| 6MWT distance (m) | | 393±118 | 367±98 | 353±78 | 0.458 |  |  |  |
| NYHA functional class (%): | | | | | | | | |
|  | I | 15 (75) | 8 (29) | 3 (15) | <0.001 |  |  |  |
| II | | 4 (20) | 15 (54) | 9 (45) |  | 0.006 | <0.001 | 0.228 |
| III | | 1 (5) | 5 (18) | 8 (40) |  |  |  |  |
| IV | | 0 | 0 | 0 |  |  |  |  |
| Aetiology: | | | | | | | | |
| Leaflet affected: | PMVL | 12 (60) | 24 (86) | 11 (55) |  |  |  |  |
|  | AMVL | 1 (5) | 1 (4) | 5 (25) | 0.037 | 0.193 | 0.282 | 0.024 |
|  | Bi-leaflet | 7 (35) | 3 (11) | 4 (20) |  |  |  |  |
| Presence of flail leaflet (%) | | 4 (20) | 6 (21) | 7 (35) | 0.564 |  | | |
| Surgical risk scores: | | | | | | | | |
| Log Euro |  | 5.6±4.7 | 4.3±3.1 | 3.8±2.4 | 0.779 |  | | |
| Log Euro II |  | 1.5±1.4 | 1.3±0.9 | 1.5±1.2 | 0.658 |  |  |  |
| STS mortality |  | 1.5±1.6 | 1.0±1.0 | 1.8±1.6 | 0.062 |  |  |  |
| STS mortality/morbidity | | 11.8±7.1 | 8.8±4.3 | 12.6±5.2 | 0.073 |  |  |  |
| Comorbidities: | | | | | | | | |
| Smoking history (%) | | 7 (35) | 12 (43) | 7 (35) | 0.857 |  | | |
| Diabetes mellitus (%) | | 2 (10) | 0 | 1 (5) | 0.263 |  |  |  |
| Hypertension (%) |  | 4 (20) | 10 (36) | 6 (30) | 0.563 |  |  |  |
| Atrial fibrillation (%) | | 4 (20) | 14 (50) | 15 (75) | 0.029 | 0.044 | 0.022 | 0.734 |
| Prior myocardial infarction (%) | | 1 (5) | 0 | 1 (5) | 0.508 |  | | |
| Prior PCI (%) |  | 2 (10) | 0 | 1 (5) | 0.263 |  |  |  |
| Prior Stroke (%) |  | 1 (5) | 0 | 0 | 0.588 |  |  |  |
| Prior TIA (%) |  | 1 (5) | 1 (4) | 1 (5) | 1 |  |  |  |
| COPD (%) |  | 2 (10) | 1 (4) | 2 (10) | 0.599 |  |  |  |
| Chronic kidney disease (%) | | 1 (5) | 0 | 1 (5) | 0.588 |  |  |  |
| Haemoglobin (g/L) | | 137±11 | 144±10 | 140±14 | 0.124 |  |  |  |
| Creatinine (umol/L) | | 79±14 | 80±18 | 86±19 | 0.386 |  |  |  |

* Duration of time until repeat CMR imaging after either surgical intervention or baseline CMR in control group. Data are mean ± standard deviation unless indicated otherwise. Abbreviations: 6MWT, 6-minute walk test; AMVL, anterior mitral valve leaflet; BMI, body mass index; BP, blood pressure; BPM, beats per minute; BSA, body surface area; COPD, chronic obstructive pulmonary disease; NYHA, New York Heart Association; PCI, percutaneous coronary intervention; PMVL; posterior mitral valve leaflet; TIA, transient ischaemic attack; STS, society of thoracic surgeons.

|  |  | Groups |  | P-values | | | |
| --- | --- | --- | --- | --- | --- | --- | --- |
|  | Control (n=20) | Repair (n=28) | Replace (n=20) | All groups | Control vs  Repair | Control vs  replace | Repair vs  Replace |
| LVEDVi (ml/m^2^) | 118±25 | 126±30 | 133±27 | 0.262 |  | | |
| LVESVi (ml/m^2^) | 50±14 | 57±21 | 62±20 | 0.132 |  |  |  |
| LVSVi (ml/m^2^) | 69±14 | 69±16 | 71±14 | 0.868 |  |  |  |
| LVEF (%) | 59±5 | 56±7.8 | 54±8.5 | 0.185 |  |  |  |
| Effective forward LVEF (%) | 36±8.3 | 28±7.9 | 27±10 | 0.004 | 0.011 | 0.009 | 1 |
| LVMi (g/m^2^) | 53±13 | 64±14 | 64±19 | 0.042 | 0.079 | 0.083 | 1 |
| LA volume indexed (ml/m^2^) | 85±23 | 93±31 | 108±37 | 0.076 |  | | |
| MR Rvol (ml) | 49±25 | 68±26 | 72±30 | 0.002 | 0.005 | 0.006 | 1 |
| MR RF (%) | 39±13 | 50±9.8 | 51±14 | 0.002 | 0.004 | 0.007 | 1 |
| RVEDVi (ml/m^2^) | 93±24 | 97±19 | 98±17 | 0.466 |  | | |
| RVESVi (ml/m^2^) | 43±12 | 53±14 | 54±17 | 0.027 | 0.056 | 0.055 | 1 |
| RVSVi (ml/m^2^) | 52±16 | 44±10 | 44±9.6 | 0.054 |  | | |
| RVEF (%) | 54±8 | 46±6.7 | 46±9.9 | 0.003 | 0.007 | 0.008 | 1 |
| AR Rvol (ml) | 3.6±3.8 | 4.2±2.1 | 3.5±2.5 | 0.176 |  | | |
| AR RF (%) | 4.8±3.9 | 6.7±3.8 | 5.9±4.3 | 0.177 |  |  |  |
| PR Rvol (ml) | 2.3±2.2 | 3.6±3.4 | 2.4±1.6 | 0.265 |  |  |  |
| PR RF (%) | 2.9±2.2 | 5.5±5.6 | 3.7±3.1 | 0.117 |  |  |  |
| TR Rvol (ml) | 12±16 | 17±15 | 14±13 | 0.24 |  |  |  |
| TR RF (%) | 13±14 | 20±18 | 16±15 | 0.201 |  |  |  |
| RAAi (cm^2^/m^2^) | 14±3 | 15±3.8 | 14±3.7 | 0.332 |  |  |  |
| Native T1 (ms)* | 1017±33 | 1028±60 | 1043±53 | 0.557 |  |  |  |
| ECV (%)* | 27.7±3.4 | 27.0±3.0 | 28.5±3.0 | 0.425 |  |  |  |
| LGE presence (%):** | 5 (28) | 10 (37) | 8 (44) | 0.639 |  |  |  |
| Non-ischaemic | 4 (22) | 7 (26) | 7 (39) | 0.795 |  |  |  |
| Ischaemic | 1 (6) | 3 (11) | 1 (6) |  |  |  |  |
| LGE (%)** | 1.0±2.0 | 3.0±4.4 | 3.3±4.3 | 0.349 |  |  |  |
| LGE (g)** | 1.1±2.3 | 3.2±4.8 | 3.7±5.1 | 0.347 |  |  |  |

*analysis performed on patients with paired baseline/follow-up data (control, n=16; repair, n=23; replace, n=18). ** analysis performed on patients with paired baseline/follow-up data (control, n=18; repair, n=27; replace, n=18). Data are mean ± standard deviation unless indicated otherwise. Abbreviations: AR, aortic regurgitation; ECV, extracellular volume; EDV, end-diastolic volume; EF, ejection fraction; ESV, end-systolic volume; i, indexed to body surface area; LA, left atrial; LGE, late gadolinium enhanced myocardium; LV, left ventricular; LVM, left ventricular mass; MR, mitral regurgitation; PR, pulmonary regurgitation; RAA, right atrial area; RF, regurgitant fraction; Rvol, regurgitant volume; RV, right ventricular; SV, stroke volume; TR, tricuspid regurgitation.

# haemodynamic and cardiac parameters from baseline to 6 month follow up assessment

|  |  | Groups |  |  | P-values | |  |
| --- | --- | --- | --- | --- | --- | --- | --- |
|  | Control (n=20 | Repair (n=28) | Replace (n=20) | All groups | Control vs  Repair | Control vs  replace | Repair vs  Replace |
| SBP (mmHg) | -0.2±21 | +0.9±11 | -0.2±12 | 0.939 |  | | |
| DBP (mmHg) | +0.5±14 | +3.1±11 | 0.0±9.2 | 0.442 |  |  |  |
| HR (bpm) | -3.0±10 | +3.1±21 | -1.0±12 | 0.432 |  |  |  |
| 6MWT distance(m) | +0.1±55 | +55±55 | +60±62 | 0.002 | 0.006 | 0.006 | 1 |
| NYHA functional class | +0.15±0.4 | -0.5±0.7 | -1.2±0.7 | <0.001 | <0.001 | <0.001 | 0.145 |
| LVEDVi (ml/m^2^) | -1.3±12 | -30±22 | -37±23 | <0.001 | <0.001 | <0.001 | 0.873 |
| LVESVi (ml/m^2^) | -1.7±7.4 | -3.5±16 | -8.3±19 | 0.382 |  | | |
| LVSVi (ml/m^2^) | -0.1±8.4 | -27±14 | -28±14 | <0.001 | <0.001 | <0.001 | 1 |
| LVEF (%) | +0.4±3.9 | -9.6±8.3 | -8.5±9.2 | <0.001 | <0.001 | 0.001 | 1 |
| Effective forward-LVEF(%) | +0.2±3.9 | +8.5±8.5 | +14±9.1 | <0.001 | <0.001 | 0.002 | 0.068 |
| LVMi (g/m^2^) | +0.3±4.3 | -4.1±10 | -3.6±11 | 0.249 |  | | |
| LA volume indexed (ml/m^2^) | +1.2±19 | -27±30 | -41±24 | <0.001 | 0.002 | <0.001 | 0.342 |
| MR Rvol (ml) | -0.1±12 | -49±20 | -62±29 | <0.001 | <0.001 | <0.001 | 0.142 |
| MR RF (%) | +0.4±7.0 | -29±11 | -40±14 | <0.001 | <0.001 | <0.001 | 0.009 |
| RVEDVi (ml/m^2^) | -0.9±5.5 | -6.2±16 | -9.8±18 | 0.160 |  | | |
| RVESVi (ml/m^2^) | +0.6±5.5 | -4.1±14 | -11±17 | 0.021 | 0.597 | 0.017 | 0.265 |
| RVSVi (ml/m^2^) | -3.3±9.0 | -2.2±11 | +0.7±8.0 | 0.545 |  | | |
| RVEF (%) | -0.8±4.0 | +0.8±9.8 | +4.9±8.2 | 0.075 |  |  |  |
| TR Rvol (ml) | +0.5±21 | -5.9±18 | -3.0±14 | 0.355 |  |  |  |
| TR RF (%) | +2.1±21 | -7.7±21 | -4.1±14 | 0.507 |  |  |  |
| RAAi (cm^2^/m^2^) | 0.0±2.5 | -0.1±2.9 | -1.0±4.1 | 0.849 |  |  |  |

Data are mean ± standard deviation. Abbreviations as in Tables A3 & A4.

# haemodynamic, and cardiac parameters at 6 month follow up assessment

|  |  | Groups |  | P-values | | | |
| --- | --- | --- | --- | --- | --- | --- | --- |
|  | Control (n=20) | Repair (n=28) | Replace (n=20) | All groups | Control  vs Repair | Control  vs replace | Repair  vs Replace |
| SBP (mmHg) | 125±14 | 127±11 | 124±15 | 0.860 |  | | |
| DBP (mmHg) | 73±10 | 80±9.6 | 76±11 | 0.066 |  |  |  |
| HR (bpm) | 68±11 | 75±16 | 72±8.4 | 0.275 |  |  |  |
| 6MWT distance (m) | 393±109 | 422±85 | 413±107 | 0.724 |  |  |  |
| NYHA functional class | 1.45±0.7 | 1.1±0.3 | 1.1±0.2 | 0.068 |  |  |  |
| LVEDVi (ml/m^2^) | 117±28 | 96±28 | 96±25 | 0.012 | 0.024 | 0.033 | 1 |
| LVESVi (ml/m^2^) | 48±15 | 53±24 | 53±21 | 0.626 |  | | |
| LVSVi (ml/m^2^) | 69±15 | 43±9.6 | 43±8.3 | <0.001 | <0.001 | <0.001 | 1 |
| LVEF (%) | 59±5 | 46±9.1 | 46±8.1 | <0.001 | <0.001 | <0.001 | 1 |
| Effective forward LVEF (%) | 36±8.9 | 36±8.9 | 41±9.2 | 0.173 |  | | |
| LVMi (g/m^2^) | 54±11 | 59±15 | 61±18 | 0.249 |  |  |  |
| LA volume indexed (ml/m^2^) | 86±28 | 66±38 | 67±28 | 0.097 |  |  |  |
| MR Rvol (ml) | 49±23 | 18±13 | 9.5±7.0 | <0.001 | <0.001 | <0.001 | 0.162 |
| MR RF (%) | 39±13 | 21±11 | 12±7.9 | <0.001 | 0.001 | <0.001 | 0.019 |
| RVEDVi (ml/m^2^) | 92±24 | 90±18 | 88±18 | 0.820 |  | | |
| RVESVi (ml/m^2^) | 44±14 | 48±13 | 44±11 | 0.303 |  |  |  |
| RVSVi (ml/m^2^) | 49±15 | 42±8.8 | 45±10 | 0.126 |  |  |  |
| RVEF (%) | 53±8 | 47±6.2 | 51±5.9 | 0.012 | 0.011 | 0.820 | 0.217 |
| TR Rvol (ml) | 13±17 | 11±11 | 11±8.6 | 0.973 |  | | |
| TR RF (%) | 15±20 | 13±11 | 12±8.7 | 0.963 |  |  |  |
| RAAi (cm^2^/m^2^) | 14±3 | 15±3.6 | 13±3.1 | 0.383 |  |  |  |
| Native T1 (ms)* | 1012±36 | 1045±42 | 1045±37 | 0.055 |  |  |  |
| ECV (%)* | 27.8±2.2 | 27.3±3.5 | 27.2±3.0 | 0.777 |  |  |  |
| LGE presence (%) **: | 7 (39) | 14 (52) | 9 (55) | 0.731 |  |  |  |
| Non-ischaemic | 5 (28) | 11 (41) | 8 (50) | 0.843 |  |  |  |
| Ischaemic | 2 (11) | 3 (11) | 1 (5) |  |  |  |  |
| LGE (%)** | 1.7±2.7 | 4.5±4.9 | 3.3±3.9 | 0.261 |  |  |  |
| LGE (g)** | 1.9±3.0 | 4.1±4.3 | 3.1±3.6 | 0.306 |  |  |  |

*analysis performed on patients with paired baseline/follow-up data (control, n=16; repair, n=23; replace, n=18). ** analysis performed on patients with paired baseline/follow-up data (control, n=18; repair, n=27; replace, n=18). Data are mean ± standard deviation unless indicated otherwise. Abbreviations as in Tables A3 & A4.
