# Supplementary material for: Cardiac reverse remodeling in primary mitral regurgitation: mitral valve replacement vs. mitral valve repair
Source: J Cardiovasc Magn Reson. 2023 Jul 27;25:43. doi: 10.1186/s12968-023-00946-9 (PMC10373289; doi:10.1186/s12968-023-00946-9)
Supplement: Supplementary file 4 — Additional file 4. Subgroup analysis of surgical groups—Comparison of baseline parameters of surgical patients when divided into groups by follow-up left ventricular ejection fraction. Subgroup analysis of surgical groups demonstrating baseline parameters between those achieving LVEF ≥ 50% vs LVEF < 50% at follow-up (Table S7). [file 12968_2023_946_MOESM4_ESM.docx]

Table S7 – Subgroup analysis of surgical groups – Comparison of baseline parameters between surgical patients when divided into groups by follow-up left ventricular ejection fraction

|  | LVEF ≥50%  (n=24) | LVEF <50% (n=28) | p-value |
| --- | --- | --- | --- |
| Age (years) | 69±8.4 | 64±11 | 0.087 |
| Male (%) | 17 (71) | 23 (82) | 0.746 |
| Duration to follow-up (days) | 191±47 | 190±27 | 0.11 |
| Systolic blood pressure (mmHg) | 127±12 | 123±15 | 0.212 |
| Diastolic blood pressure (mmHg) | 78±12 | 76±12 | 0.437 |
| Aetiology: | | | |
| Leaflet affected (%): PMVL | 20 (83) | 18 (64) | 0.539 |
| AMVL | 3 (13) | 3 (11) | 1 |
| Bi-leaflet | 6 (25) | 2 (7) | 0.278 |
| Presence of flail leaflet (%) | 7 (29) | 8 (29) | 0.54 |
| Comorbidities: | | | |
| Smoking history (%) | 11 (38) | 11 (39) | 0.576 |
| Diabetes mellitus (%) | 1 (4) | 1 (4) | 1 |
| Hypertension (%) | 8 (33) | 9 (32) | 1 |
| Atrial fibrillation (%) | 14 (58) | 15 (54) | 0.269 |
| Prior myocardial infarction (%) | 1 (4) | 0 | 0.442 |
| Prior Stroke (%) | 0 | 0 | n/a |
| Prior TIA (%) | 0 | 2 (7) | 0.497 |
| COPD (%) | 2 (8) | 1 (5) | 0.577 |
| Chronic kidney disease (%) | 0 | 1 (5) | 1 |
| Haemoglobin (g/L) | 141±13 | 143±11 | 0.526 |
| Creatinine (μmol/L) | 81±15 | 86±22 | 0.386 |
| Functional parameters: | | | |
| 6MWT distance (m) | 368±94 | 356±84 | 0.636 |
| NYHA class | 2.2±0.7 | 2.5±0.7 | 0.629 |
| Surgical Parameters: | | | |
| Mitral valve replacement (%) | 10 (42) | 12 (43) | 1 |
| Coronary artery bypass grafting (%) | 3 (13) | 1 (4) | 0.31 |
| CMR parameters: | | | |
| LVEDVi (ml/m^2^) | 112±23 | 139±29 | 0.001 |
| LVESVi (ml/m^2^) | 47±12 | 67±21 | <0.001 |
| LVSVi (ml/m^2^) | 66±15 | 72±15 | 0.173 |
| LVEF (%) | 59±6.2 | 52±8.0 | 0.002 |
| Effective forward LVEF | 29±8.3 | 26±8.9 | 0.487 |
| LVMi (g/m^2^) | 56±14 | 69±15 | 0.196 |
| LA volume indexed (ml/m^2^) | 93±28 | 105±37 | 0.347 |
| MR Rvol (ml) | 60±25 | 75±28 | 0.028 |
| MR RF (%) | 47±11 | 54±10 | 0.028 |
| RVEDVi (ml/m^2^) | 91±21 | 101±16 | 0.059 |
| RVESVi (ml/m^2^) | 48±15 | 57±13 | 0.013 |
| RVSVi (ml/m^2^) | 43±11 | 44±8.8 | 0.721 |
| RVEF (%) | 48±8.3 | 44±7.1 | 0.08 |
| Native T1 (ms)* | 1026±48 | 1037±63 | 0.679 |
| ECV (%)* | 27.7±3.5 | 27.5±2.8 | 0.723 |
| LGE presence (%): ** | 9 (41) | 12 (43) | 1 |
| non-ischaemic | 9 (41) | 8 (30) | 0.167 |
| ischaemic | 0 | 4 (14) |  |
| LGE (%)*** | 3.1±4.5 | 3.2±4.1 | 0.687 |
| LGE (g)*** | 3.2±4.5 | 3.6±5.1 | 0.687 |

*analysis performed on available baseline data (LVEF≥50%, n=20; LVEF<50%, n=25). ** analysis performed on available baseline data (LVEF≥50%, n=22; LVEF<50%, n=28). *** analysis performed on available baseline data (LVEF≥50%, n=22; LVEF<50%, n=27). Data are mean ± standard deviation unless indicated otherwise. Abbreviations: 6MWT, 6-minute walk test; AMVL, anterior mitral valve leaflet; BP, blood pressure; CABG; coronary artery bypass grafting; COPD, chronic obstructive pulmonary disease; ECV, extracellular volume; EDV, end-diastolic volume; EF, ejection fraction; ESV, end-systolic volume ; i, indexed to body surface area; LA, left atrial; LGE, late gadolinium enhanced myocardium; LV, left ventricular; LVM, left ventricular mass; MR, mitral regurgitation ; MVR, Mitral valve replacement; NYHA, New York Heart Association PMVL; posterior mitral valve leaflet; RF, regurgitant fraction; Rvol, regurgitant volume; RV, right ventricular; SV, stroke volume; TIA, transient ischaemic attack.
