# Supplementary material for: Cardiac reverse remodeling in primary mitral regurgitation: mitral valve replacement vs. mitral valve repair
Source: J Cardiovasc Magn Reson. 2023 Jul 27;25:43. doi: 10.1186/s12968-023-00946-9 (PMC10373289; doi:10.1186/s12968-023-00946-9)
Supplement: Supplementary file 5 — Additional file 5. Subgroup analysis of surgical groups by baseline presence or absence of late gadolinium enhanced myocardium. Subgroup analysis of surgical groups comparing those with and without late gadolinium enhancement at baseline—baseline patient characteristics and surgical parameters (Table S8) and baseline, change and follow-up CMR and functional parameters (Table S9). [file 12968_2023_946_MOESM5_ESM.docx]

# Table S8 – Subgroup analysis of surgical groups by baseline presence (LGE+) or absence (LGE-) of late gadolinium enhanced myocardium - Baseline patient characteristics and surgical parameters

|  | LGE- (n=29) | LGE+ (n=21) | p-value |
| --- | --- | --- | --- |
| Age (years) | 64±10 | 65±8.1 | 0.106 |
| Male (%) | 21 (72) | 18 (86) | 0.319 |
| Duration to follow-up (days) | 195±30 | 185±47 | 0.644 |
| Systolic BP (mmHg) | 124±14 | 121±13 | 0.405 |
| Diastolic BP (mmHg) | 76±11 | 75±13 | 0.452 |
| Aetiology: | | | |
| Leaflet affected (%): PMVL prolapse | 20 (69) | 16 (76) | 0.752 |
| AMVL prolapse | 5 (17) | 1 (5) | 0.38 |
| Bi-leaflet | 4 (14) | 4 (19) | 0.706 |
| Presence of flail leaflet (%) | 10 (34) | 4 (19) | 0.341 |
| Comorbidites: | | | |
| Smoking history (%) | 11 (38) | 11 (52) | 0.391 |
| Diabetes mellitus (%) | 0 | 1 (5) | 0.42 |
| Hypertension (%) | 10 (34) | 6 (29) | 0.763 |
| Atrial fibrillation (%) | 14 (48) | 14 (67) | 0.254 |
| Prior myocardial infarction (%) | 1 (3) | 0 | 1 |
| Prior Stroke (%) | 0 | 0 | 0.503 |
| Prior TIA (%) | 0 | 0 | 0.503 |
| COPD (%) | 2 (7) | 1 (5) | 1 |
| Chronic kidney disease (%) | 0 | 1 (5) | 0.42 |
| Surgical Parameters: | | | |
| Mitral valve replacement (%) | 10 (34) | 10 (48) | 0.393 |
| Coronary artery bypass grafting (%) | 1 (3) | 3 (14) | 0.297 |

Data are mean ± standard deviation unless indicated otherwise. Abbreviations: 6MWT, 6-minute walk test; AMVL, anterior mitral valve leaflet; BP, blood pressure; COPD, chronic obstructive pulmonary disease; LGE+, late gadolinium enhancement present at baseline; LGE-, late gadolinium enhancement absent at baseline; NYHA, New York Heart Association; PMVL; posterior mitral valve leaflet; TIA, transient ischaemic attack.

# Table S9 – Subgroup analysis of surgical groups by baseline presence (LGE+) or absence (LGE-) of late gadolinium enhanced myocardium - Baseline, change and follow-up CMR and functional parameters

| CMR parameters: |  | Baseline: |  |  | Change: |  | Follow-up: | |  |
| --- | --- | --- | --- | --- | --- | --- | --- | --- | --- |
|  | LGE - (n=29) | LGE + (n=21) | p- value | LGE - (n=29) | LGE+ (n=21) | p- value | LGE- (n=29) | LGE+ (n=21) | p- value |
| LVEDVi (ml/m^2^) | 127±32 | 120±25 | 0.685 | -32±20 | -32±23 | 0.979 | 95±27 | 88±24 | 0.776 |
| LVESVi (ml/m^2^) | 56±21 | 57±19 | 0.761 | -4.9±15 | -6.7±19 | 0.644 | 52±22 | 50±21 | 0.658 |
| LVSVi (ml/m^2^) | 71±16 | 63±13 | 0.189 | -27±14 | -25±14 | 0.554 | 43±14 | 38±16 | 0.341 |
| LVEF (%) | 56±7 | 51±8.7 | 0.169 | -9.0±8.0 | -8.7±9.7 | 0.716 | 47±8.7 | 42±8.6 | 0.326 |
| Effective forward LVEF (%) | 29±8.3 | 26±8.9 | 0.147 | +10±9.1 | +11±7.9 | 0.784 | 39±10 | 37±6.7 | 0.376 |
| LVMi (g/m^2^) | 61±16 | 63±14 | 0.291 | -3.1.±9.5 | -3.6±12 | 0.84 | 58±16 | 60±14 | 0.35 |
| LA volume (ml/m^2^) | 94±31 | 100±35 | 0.347 | -27±28 | -36±27 | 0.318 | 67±39 | 65±26 | 0.498 |
| MR Rvol (ml) | 67±28 | 66±26 | 0.86 | -52±21 | -53±29 | 0.976 | 15±13 | 13±9.6 | 0.937 |
| MR RF (%) | 49±11 | 51±12 | 0.204 | -31±10 | -35±16 | 0.21 | 18±12 | 16±8.7 | 0.84 |
| RVEDVi (ml/m^2^) | 98±21 | 90±16 | 0.413 | -7.5±17 | -4.6±20 | 0.513 | 90±18 | 86±20 | 0.761 |
| RVESVi (ml/m^2^) | 53±18 | 50±10 | 0.875 | -5.7±16 | -5.7±14 | 0.404 | 47±14 | 45±11 | 0.945 |
| RVSVi (ml/m^2^) | 45±10 | 40±10 | 0.184 | -1.8±10 | +1.0±10 | 0.281 | 43±8.4 | 41±11 | 0.85 |
| RVEF (%) | 47±8.9 | 42±6.0 | 0.024 | +1.4±10 | +3.4±6.8 | 0.355 | 48±6.8 | 45±5.2 | 0.713 |
| Functional indices: | | | | | | | | | |
| NYHA functional class | 2.0±0.7 | 2.1±0.7 | 0.7 | -1.0±0.7 | -1.0±0.8 | 0.567 | 1.1±0.3 | 1.1±0.3 | 0.638 |
| 6MWT (m) | 364±87 | 344±94 | 0.575 | +64±47 | +52±83 | 0.752 | 428±97 | 398±91 | 0.397 |

Data are mean ± standard deviation. Abbreviations: 6MWT, 6-minute walk test distance; CMR, cardiovascular magnetic resonance; EDV, end-diastolic volume; EF, ejection fraction; ESV, end-systolic volume; i, indexed to body surface area; LA, left atrial; LGE+, late gadolinium enhancement present at baseline; LGE-, late gadolinium enhancement absent at baseline; ; LV, left ventricular; LVM, left ventricular mass; MR, mitral regurgitation; NYHA, New York Heart Association; RF, regurgitant fraction; Rvol, regurgitant volume; RV, right ventricular; SV, stroke volume.
